# The role of two major nucleoid-associated proteins in Streptomyces, HupA and HupS, in stress survival and gene expression regulation

**DOI:** 10.1186/s12934-024-02549-0

**Published:** 2024-10-14

**Authors:** Agnieszka Strzałka, Jakub Mikołajczyk, Klaudia Kowalska, Michał Skurczyński, Neil A. Holmes, Dagmara Jakimowicz

**Affiliations:** 1 Molecular Microbiology Department, Faculty of Biotechnology, University of Wroclaw, Wroclaw, Poland; 2 The John Innes Centre, Norwich Research Park, Norwich, NR4 7UH UK

**Keywords:** Streptomyces, Nucleoid-associated proteins (NAPs), HU, Gene expression regulation, Stress response, Secondary metabolite gene cluster

## Abstract

**Background:**

*Streptomyces* are sporulating soil bacteria with enormous potential for secondary metabolites biosynthesis. Regulatory networks governing *Streptomyces coelicolor* differentiation and secondary metabolites production are complex and composed of numerous regulatory proteins ranging from specific transcriptional regulators to sigma factors. Nucleoid-associated proteins (NAPs) are also believed to contribute to regulation of gene expression. Upon DNA binding, these proteins impact DNA accessibility. Among NAPs, HU proteins are the most widespread and abundant. Unlike other bacteria, the *Streptomyces* genomes encode two HU homologs: HupA and HupS, which differ in structure and expression profile. However, it remained unclear whether the functions of both homologs overlap. Additionally, although both proteins have been shown to bind the chromosome, their rolesin transcriptional regulation have not been studied so far.

**Results:**

In this study, we explore whether HupA and HupS affect *S. coelicolor* growth under optimal and stressful conditions and how they control global gene expression. By testing both single and double mutants, we address the question of the complementarity of both HU homologs. We show that the lack of both *hup* genes led to growth and sporulation inhibition, as well as increased spore fragility. We also demonstrate that both HU homologs can be considered global transcriptional regulators, influencing expression of between 2% and 6% genes encoding among others proteins linked to global regulatory networks and secondary metabolite production.

**Conclusions:**

We identify the independent HupA and HupS regulons, as well as genes under the control of both HupA and HupS proteins. Our data indicate a partial overlap between the functions of HupA and HupS during *S. coelicolor* growth. HupA and HupS play important roles in *Streptomyces* regulatory network and impact secondary metabolite clusters.

**Supplementary Information:**

The online version contains supplementary material available at 10.1186/s12934-024-02549-0.

## Background

Nucleoid-associated proteins (NAPs) are bacterial proteins that perform a role similar to eukaryotic histones. By coating, bridging and bending DNA molecules, these proteins organize and compact DNA. They also share other properties with histones, such as small size, a high content of basic amino acids, and a lack of (or very low) DNA sequence specificity. What is more, similarly to histones, by modifying the accessibility of DNA to transcriptional machineries, they play a role in the regulation of gene expression [1, 2].

In many bacterial cells, the most abundant NAP is a small, positively charged protein HU (Heat Unstable) [3]. In *Escherichia coli*, HU abundance, estimated to be between 30,000 and 55,000 HU molecules per cell, reaches its peak during exponential growth [4]. Like other NAPs, this protein exhibits little sequence specificity, but shows increased affinity to supercoiled, single stranded or distorted DNA [5–7]. Interestingly, the impact of HU binding on DNA structure in vitro depends on protein/DNA molar ratio and osmolarity. At low salt concentration, HU promotes DNA compaction, while at high salt concentration, its binding leads to the formation of “rigid filaments” [8–10]. In vivo HU homologs affect gene expression and change the distribution of RNA polymerase by altering DNA topology within promoter regions or promoting long-range DNA contacts [11, 12]. Recent studies have revealed that HU homologs influence transcription of genes connected to stress response or virulence in many bacterial species including: *E. coli* [13, 14] *Salmonella enterica* [15], *Vibrio parahaemolyticus* [16], *Francisella tularensis* [17] and *Helicobacter pylori* [18].

Most of the Proteobacteria and Bacteroidetes, like *E. coli*, possess two HU homologs that form homo- or heterodimers, while other phyla have only one HU homolog which forms a homodimer [19]. In *E. coli* two HU homologs, HUα and HUβ, share 69% amino acid identity [20], but differ in affinity for different DNA structures, binding modes and their level during culture growth [5, 21, 22]. Notably, actinobacterial HU homologs form a distinct group and are characterized by the presence of a long, positively charged C-terminal domain [23]. In mycobacteria, the presence of the C-terminal domain is necessary for DNA binding [24, 25]. Interestingly, a few actinobacterial orders, namely *Streptomycetales*, *Propionibacteriales*, *Kineosporiales and Micrococcales*, possess two HU homologs differing in structure: one with an extended C-terminal domain and one similar to *E. coli* HU.

*Streptomyces* are soil-dwelling bacteria known for antibiotic production and a complex life cycle that includes sporulation. The *Streptomyces coelicolor* long, linear chromosome (8.6 Mbp) contains more than 20 biosynthetic gene clusters involved in production of secondary metabolites [26]. Most of them remain inactive during growth, while a few are activated before the start of sporulation [27]. The structure of the *Streptomyces* chromosome also changes during growth, from uncondensed in vegetative hyphae to tightly packaged in unigenomic spores or late stationary phase [28, 29]. These changes of *Streptomyces* chromosome organisation seem to be related, among other factors, to changes in the levels of two HU homologs. According to proteomic and transcriptomic data, HupA is the most abundant NAP during vegetative growth [30, 31] and binds preferentially at the central region of the chromosome without detectable sequence specificity [32]. HupS levels are relatively low during vegetative growth, but they increase significantly during sporulation and the protein shows enhanced binding in terminal regions of the chromosome and very weak sequence specificity [29, 33, 34]. In contrast to *E. coli* HU homologs, HupA and HupS share little sequence homology. HupA is more similar to the canonical *E. coli* HU, but has only 38% identity with N-terminal domain of HupS. The long positively charged C-terminal domain of HupS contains multiple lysine repeats, similar to those found in other Actinobacterial proteins such as topoisomerase I (TopA) [30, 33, 35, 36]. Interestingly, the lack of only one HU homolog moderately affects *Streptomyces* growth. Deletion of *hupA* in both *S. coelicolor* and *S. lividans* reduced their growth rate [32, 37], while *hupS* deletion in *S. coelicolor* and *S. venezuelae* resulted in diminished compaction of chromosomes in spores, which were more sensitive to high temperatures than wild type spores [29, 33]. Apart from HupA and HupS, *Streptomyces* possess several other nucleoid-associated proteins such as sIHF, DpsA, Lsr2 or a novel protein, Gbn, which contribute to nucleoid structure maintenance and participate in the regulation of *Streptomyces* metabolism and development [38–41].

Given the minimal phenotype of either *hupA* or *hupS* deletion mutants, we expected that the functions of HupA and HupS may be partially complementary. The cooperation between HupA and HupS has not yet been described. Thus, in this work we sought to examine the consequences of *hupA* and *hupS* deletion for *S. coelicolor* growth under optimal and stressful conditions. Given that NAPs binding impacts transcription, the phenotype of *hupS* and *hupA* mutant strains could be at least partially attributed to their influence on transcription. Therefore, we also set out to establish HupA and HupS regulons in *S. coelicolor*. We show that both HU homologs regulate genes involved in secondary metabolism and stress response. Comparison of HupA and HupS regulons allowed us to determine the extent of HupA and HupS cooperation. Taken together, our results suggest that HupA and HupS binding has an impact on global gene expression, facilitating survival under various environmental conditions.

## Methods

### Growth conditions and genetic modifications of bacterial strains

The *E. coli* and *S. coelicolor* strains used are listed in Supplementary Table S1. The culture conditions, antibiotic concentrations, and transformation and conjugation methods followed the general procedures for *E. coli* [42] and *Streptomyces* [43]. For plate cultures of *S. coelicolor* strains, minimal medium supplemented with 1% mannitol (MM) or Soya Flour Mannitol medium (SFM) was used. For liquid cultures YEME/TSB medium was used which is 1:1 ratio mix of YEME and Tryptic Soy Broth (TSB) medium. For the growth rate evaluation, *S. coelicolor* cultures in YEME/TSB or ’79’ medium were inoculated with spores to final 0.01 U/ml (1 U of spores increases medium absorbance by 1) and cultured in microplates (250 µl per well), for 72 h at 30 °C using a Bioscreen C (Automated Growth Curves Analysis System, Growth Curves USA), with five experimental replicates for each strain. Absorbance was measured for 600 nm. *S. coelicolor* growth curves were analyzed using the drc R package, for each strain the half-time was determined by the logistic model.

In order to construct a strain lacking *hupA* and *hupS* genes plasmid pKF289 [33] was introduced into ASMK011 strain (*ΔhupA::scar*). After conjugation colonies resistant to hygromycin were obtained yielding strain ASMK019 (*ΔhupA::scar ΔhupS::higro*), which was verified using PCR. To create a complementation strain, the *hupA* gene along with its native promoter was amplified using the hupA_pSET_FW and hupA_pSET_RV primers, and subsequently ligated into the pSET152 vector. Obtained was plasmid was introduced into ASMK019 strain. After conjugation colonies resistant to hygromycin and apramycin were obtained yielding strain ASMK019.2 (*ΔhupA::scar ΔhupS::higro* pSET152 *hupA*). DNA manipulations were carried out by standard protocols [42]. The genetic modifications of the obtained strains were verified by PCR and sequencing. The oligonucleotides used for PCR are listed in Supplementary Table S2.

### Stress sensitivity analyses

First, spore concentrations were measured using a Thoma Cell Counting Chamber and Leica DM6 B fluorescence microscope equipped with a 40x objective. Spores were subjected to either increased temperature (60 °C for 15–45 min) or detergent (2.5–10% SDS for 1 h in room temperature). Next serial dilutions of spores were plated on SFM medium. To test UV sensitivity, spores were first plated and then exposed to UV light for 15–45 s. For oxidative stress analysis serial dilutions of spores were plated on the SFM medium containing increasing concentrations of H_2_O_2_ (0–1 mM) and incubated at 30 °C for 5 days. After 5 days of incubation at 30 °C the number of growing colonies was counted to determine the percentage of plated spores that survived the stress.

### Microscopy analysis

For microscopy analysis *S. coelicolor* spores were cultured on microscopy coverslips inserted at a 45° angle in a MM solid medium containing 1% mannitol. After 44 h mycelia were fixed with a 2.8% paraformaldehyde/0.00875% glutaraldehyde mixture for 10 min at room temperature. After fixation samples were digested with lysozyme (2 mg/ml in 20 mM Tris–HCl supplemented with 10 mM EDTA and 0.9% glucose) for 2 min, washed with PBS, blocked with 2% BSA in a PBS buffer for 10 min and incubated with 0.1–1 µg/ml DAPI (4’,6-diamidyno-2-fenyloindol, Molecular Probes) and 1–10 µg/ml WGA-Texas Red (Wheat Germ Agglutinin-Texas Red) for 60 min. Fluorescence microscopy was performed using a Leica DM6 B fluorescence microscope equipped with a 100x oil immersion objective. Sporulating hyphae were analyzed using custom protocols involving *Fiji* [44] and *R* software [45], the code is available at https://github.com/astrzalka/sporecounter.

### RNA isolation

For RNA-seq, total RNA was isolated from 30 ml YEME/TSB cultures. Cultures were inoculated with *S. coelicolor* spores (the amount of spores was normalized by OD measurement of preliminary cultures), and cultivated in flasks with spring coils for 24–36 h at 30 °C. For the osmotic stress experiment growth medium was supplemented with NaCl to a final concentration 0.5 M. Mycelia were collected at two time points (mid-log and early stationary phase, determined individually for each strain based on the growth curve) by collecting 2 ml from the culture and centrifugation. Cell pellets were frozen and stored at -70 °C for subsequent RNA isolation. RNA was isolated using the RNeasy Mini Kit (Qiagen) following manufacturer’s instructions, DNA digestions were performed using on column digestion with DNase-I (Qiagen) and TURBO DNase I (Ambion). RNA quality and concentration was measured using Nanodrop 1000 (Thermo Fisher Scientific) and Qubit 4 fluorometer (Thermo Fisher Scientific). The absence of DNA in the sample was confirmed using PCR.

### RNA-seq bioinformatic analysis

Library preparation and RNA-sequencing was performed by Genewiz (Germany). Trimmomatic software (version 0.39) [46] with parameters minlen: 30, leading: 3, and trailing: 3, was used to remove illuminaclip adapter sequences from sequenced reads. Obtained reads were mapped to *S. coelicolor* genome (NC_003888.3) using *Bowtie2* software (version 2.3.5.1) [47, 48] and processed with *samtools* (version 1.10) using default parameters [49]. On average 4 × 10^6^ reads mapped successfully to the *S. coelicolor* genome. Differential analysis was performed using R packages *Rsubread* (version 2.10) and *edgeR* (version 3.38) [50, 51] following a protocol described in [52]. The gene count matrix was normalized using *egdeR* and a quasi-likelihood negative binomial was fitted to the data. Differential expression was tested using *glmTtreat* function with a 1.5 fold change threshold. Genes were considered to be differentially expressed if the false discovery rate (FDR) was below 0.05 threshold and Log_2_FC value was above 1.5. For data visualization *ggplot2* (version 3.3.6) [53], *ggVennDiagram* (version 1.2.0) [54] and *tidyHeatmap* (version 1.6.0) [55] R packages were used. Heatmaps show normalized log_2_ of CPM values. Cluster analysis was performed using the *clust* programme (version 1.10.8) [56]. RNA isolation and Reverse-Transcription and Quantitative PCR (RT-qPCR).

### RT-PCR

RNA for RT-qPCR was isolated from 5 mL YEME/TSB liquid medium *S. coelicolor* cultures cultivated for 24–36 h. Mycelia were collected by centrifugation, frozen and stored at -70 °C for subsequent RNA isolation. RNA was isolated using the RNeasy Mini Kit (Qiagen) following manufacturer’s instructions, digested with TURBO DNase I (Invitrogen) and checked for chromosomal DNA contamination using PCR. A total of 500 ng of RNA was used for cDNA synthesis using the Maxima First Strand cDNA synthesis kit (Thermo Fisher Scientific) in a final volume of 20 µl. Obtained cDNA was diluted to 100 µl and directly used for quantitative PCRs performed with PowerUp SYBR Green Master Mix (Applied Biosystems). The relative level of the transcript of interest was quantified using the comparative ΔΔCt method using the *hrdB* transcript as the endogenous control (StepOne Plus real-time PCR system, Applied Biosystems).

## Results and discussion

### Deletion of *hupA* and *hupS* has a synergistic effect on *Streptomyces* growth and development

Previous reports concerning the role of HU homologs in *Streptomyces* showed only a moderate phenotype of deletion strains; specifically: the Δ*hupA* mutant exhibited slower growth [32, 37], while the Δ*hupS* mutant displayed decreased nucleoid compaction and increased spore sensitivity to thermal stress [29, 33]. Given the presence of both protein in the cell, albeit at significantly different levels, we wondered if the functions of HU homologs could be redundant in *Streptomyces* and whether HupA or HupS would compensate for the loss of the other homolog. To test this hypothesis, we constructed a double deletion Δ*hupA*Δ*hupS S. coelicolor* strain and compared its growth under various conditions to that of the Δ*hupA*, Δ*hupS* and wild type (M145) strains.

**Fig. 1:**
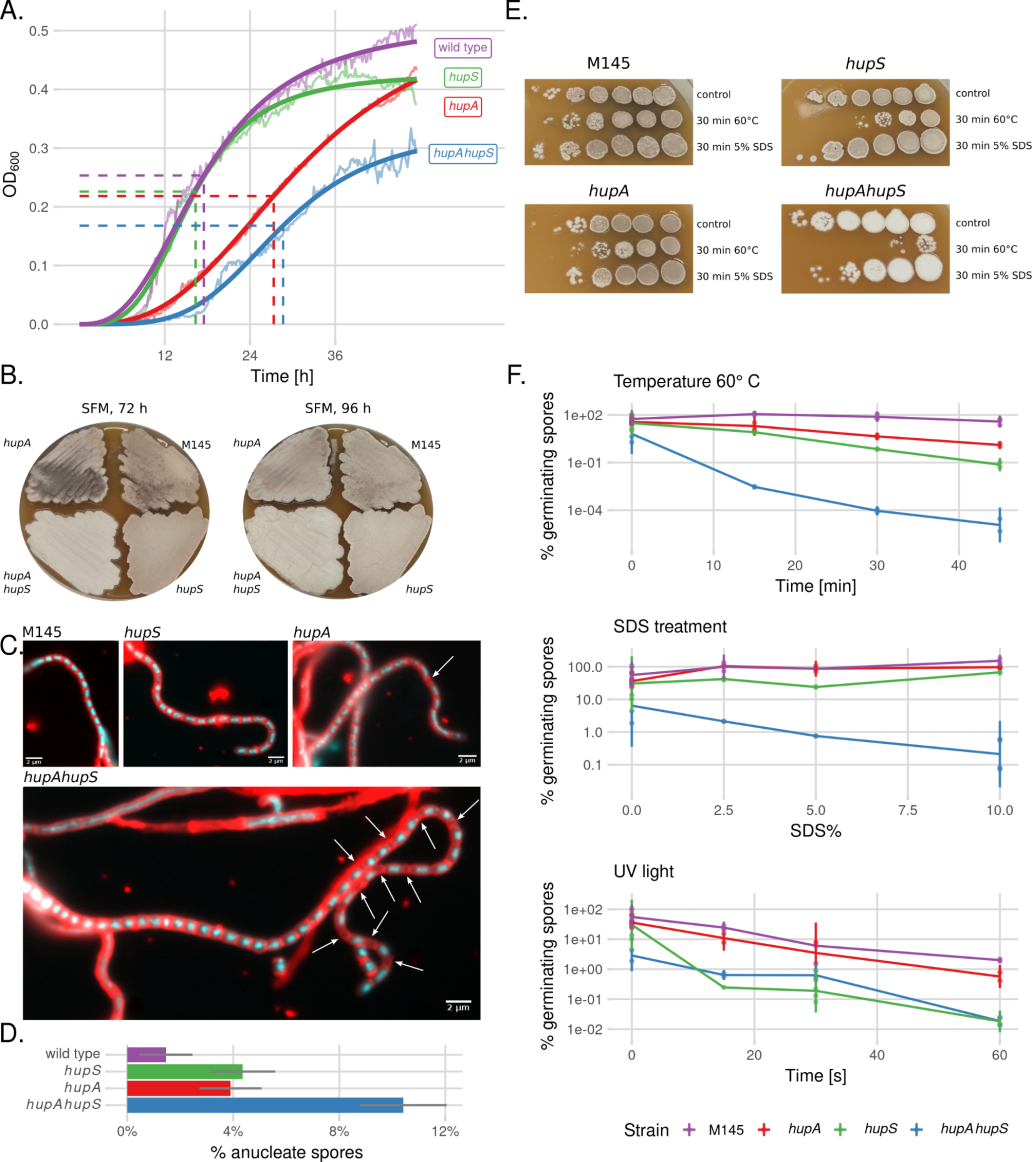
Deletion *hupA* or/and *hupS* inhibits growth and increases stress sensitivity of *S. coelicolor* spores **A.** Growth curves of wild type (purple), Δ*hupA* (red), *ΔhupS* (green) and Δ*hupAΔhupS* (blue) *s* trains in liquid ‘79’ medium. Bold lines represent logistic model while striped lines show calculated half time calculated. **B.** Colonies of wild type, Δ*hupA*, Δ*hupS* and Δ*hupA*Δ*hupS s* trains on solid SFM medium after incubation in 30 °C for 72 and 96 h. Grey appearance of colonies indicates sporulation. **C.** Microscopic images of wild type, Δ*hupA*, *ΔhupS* and Δ*hupA*Δ*hupS* hyphae stained with DAPI (blue, DNA) and WGA-Texas Red (red, cell wall). White arrows indicate positions of anucleate prespore compartments. Scale bar 2 μm. **D.** Percentage of anucleate spores in sporulating hyphae of wild type (purple, 547 spores), Δ*hupA* (red, 1051 spores), Δ*hupS* (green, 1077 spores) and Δ*hupA*Δ*hupS* (blue, 1344 spores) *s* trains. Grey lines represent 95% confidence interval. **E.** Growth of wild type, Δ*hupA*, Δ*hupS* and Δ*hupA*Δ*hupS s* trains after exposure of spores to high temperature (60 °C) or detergent (5% SDS) for 30 min before plating. Serial dilutions of all strains were plated on solid SFM medium. **F.** Percentage of germinating spores of wild type (purple), Δ*hupA* (red), *ΔhupS* (green) and Δ*hupAΔhupS* (blue) *s* trains after exposure to high temperature (60 °C), detergent (5% SDS) or UV light. Lines show mean values while errorbars represent standard deviation

The growth of Δ*hupA*Δ*hupS* strain was more inhibited than that of either of single mutant (Fig. 1A). Both the Δ*hupA*Δ*hupS* and Δ*hupA* strains showed a significant delay in the initiation of growth in liquid medium (half time 28.7 and 27.3 h, based on a logistic growth model, respectively) compared to wildtype and Δ*hupS* strains (17.5 and 16.3 h, respectively). However, after the complementation of the double deletion strain with *hupA* gene, an improvement in growth was observed (Fig. S1 A). A similar growth delay was also observed for the Δ*hupA*Δ*hupS* strain during culture on solid medium (Fig. 1B). Moreover, Δ*hupA*Δ*hupS* colonies remained white, as this strain did not produce the grey-brown spore pigment characteristic for *S. coelicolor* (Fig. 1B). Microscopic observations confirmed, however, that the double deletion strain was able to sporulate (Fig. S1 B), but only after a prolonged incubation (~ 7 days) compared to 3 days for the wild type and Δ*hupS* strains, and 4 days for the Δ*hupA* strain. The analysis of sporogenic hyphae in the mutant strains also showed that the Δ*hupA*Δ*hupS* strain had chromosome segregation defects, with 10.4% of spores lacking DNA; in comparison, the wild type strain had only 1.5% anucleate spores. Segregation defects were detectable also in single deletion strains: 3.9% spores lacked DNA in the Δ*hupA* strain and 4.4% in the Δ*hupS* strain (Fig. 1C, D).

Given the chromosome segregation defects and the lack of spore pigmentation in the Δ*hupA*Δ*hupS* strain, we expected that its spores would be less viable than those of Δ*hupA* or Δ*hupS* spores. Indeed, spores from the Δ*hupA*Δ*hupS* strain were significantly less resistant to all tested stress factors: high temperature, presence of SDS as well as exposure to UV light or hydrogen dioxide. Colony forming unit (CFU) calculations showed that less than 0.00001% of Δ*hupA*Δ*hupS* spores survived a 45 min incubation at 60 °C compared to survival of 40.5% of wild type spores and 1.3% and 0.07% of Δ*hupA* and Δ*hupS* spores, respectively. Treatment with SDS affected solely the spores of the Δ*hupA*Δ*hupS* strain (less than 1% spores germinated), while for wild type and single deletion strains, treatment with SDS increased the germination rate from 60% to around 100% (Fig. 1E, F). UV light exposure for 60 s resulted in the survival of less than 0.1% of Δ*hupS* and Δ*hupA*Δ*hupS* spores, whereas spores of *ΔhupA* and wild type strains were less affected and 0.6 and 2% of the spores still formed colonies, respectively (Fig. 1F). The presence of H_2_O_2_ in both liquid and solid medium strongly inhibited not only germination but also growth of Δ*hupS* and Δ*hupA*Δ*hupS* strains, while the Δ*hupA* strain tolerated higher concentrations of H_2_O_2_. The H_2_O_2_ concentration of 1.75 mM led to growth inhibition of Δ*hupA* strain but not of the wild type strain (Fig. S2). Thus, factors causing DNA damage such as UV light or hydrogen peroxide, were found to be particularly harmful to spores of strains lacking HupS.

In summary, the deletion of both genes encoding HU homologs, *hupA* and *hupS*, in *S. coelicolor* resulted in a more severe phenotype than that of the single deletion mutants: more pronounced growth retardation, chromosome segregation defects and changes in colony pigmentation. Severity of the Δ*hupA*Δ*hupS* strain phenotype compared to single deletion mutants suggested an overlap between HupA and HupS functions. The absence of both HU homologs decreased also spores resistance to stress conditions. The elevated sensitivity to some of the stress factors, such as UV light or reactive oxygen species, could be explained either by lack of the physical protection of DNA or by transcriptional changes of genes involved in stress response.

### HupA and HupS regulons partially overlap

Given that a diminished stress response may result from the impact of HU homologs on gene expression, we set out to test whether the elimination of *hupA* or *hupS* would lead to transcriptional changes in *S. coelicolor*. Since the lack of HupA and HupS in *S. coelicolor* resulted in somewhat different phenotypes, we expected that their regulatory networks may not entirely overlap. To establish the HupA and HupS regulons and compare them to transcriptional changes in the double mutant strain, we performed an RNA-seq experiment for the Δ*hupA*, Δ*hupS* and Δ*hupA*Δ*hupS* strains, in comparison to a wild type control, all grown in liquid YEME/TSB medium. For each strain two timepoints were chosen based on the growth curves (Fig. S3): the middle of exponential growth (mid-log) (20 h for wild type strain, Δ*hupA* and Δ*hupS*, 24 h for Δ*hupA*Δ*hupS* strain) and the early stationary phase of growth (26 h for wild type strain, Δ*hupA* and Δ*hupS*, 30 h for Δ*hupA*Δ*hupS* strain). Differential expression analysis of all *S. coelicolor* genes using the edgeR package determined which genes were affected by either *hupA* or *hupS* deletions when compared to the wild type strain at the two tested time points (Table S3).

**Fig. 2:**
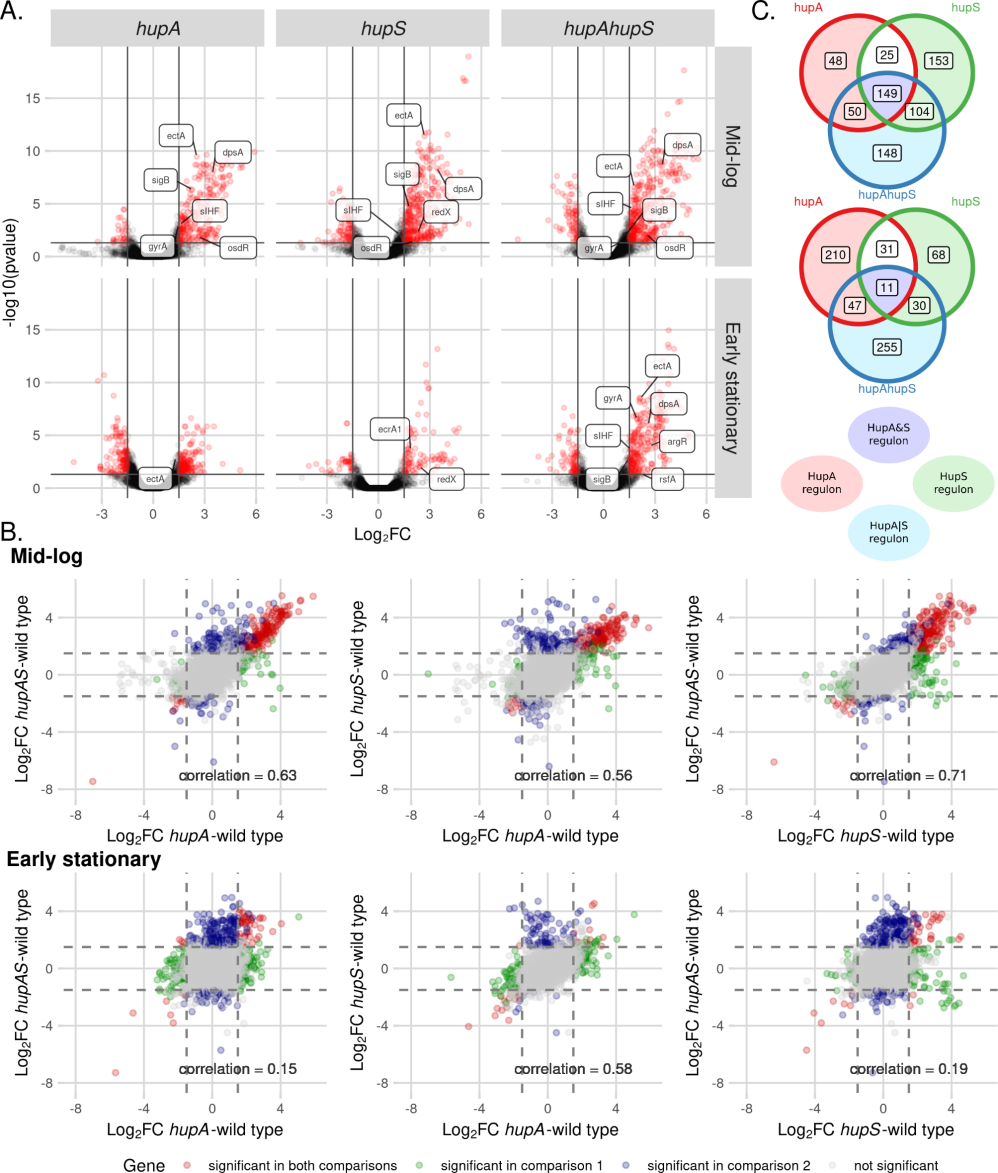
Global changes of gene expression in Δ*hupA*, Δ*hupS* and Δ*hupA*Δ*hupS* strains **(A)** Volcano plots showing altered gene expression in Δ*hupA*, *ΔhupS* and Δ*hupA*Δ*hupS* strains at mid-log and early stationary phase compared to the wild type strain. For each strain significantly changes genes (FDR ≤ 0.05, |Log_2_FC| > 1.5) are shown in red. Chosen differentially expressed genes are labelled. **(B)** Venn diagrams showing significantly changed genes unique and common for analyzed strains; Δ*hupA*, *ΔhupS* and Δ*hupA*Δ*hupS* during mid-log and early stationary phase. **(C)** Scatterplots showing correlation between Δ*hupA*, Δ*hupS* and Δ*hupA*Δ*hupS* transcriptomes from mid-log and early stationary phase compared to the wild type strain. Pearson correlation coefficient is shown on each plot. Genes found to be significant in both comparisons (FDR ≤ 0.05, |Log_2_FC| > 1.5) are shown in red, while genes significant in only one of the comparisons are marked as blue or green, grey dots represent genes not affected by *hupA* and/or *hupS* deletions

The number of genes whose transcription changed in D *hupA* was similar during mid-log and early stationary phase (272 and 299 genes, respectively), while *hupS* deletion affected expression of more genes during mid-log than in the early stationary phase (431 and 140, respectively) (Fig. 2A, B). Gene expression was most altered in the double deletion mutant, with 451 genes changed during mid-log and 343 genes during the early stationary phase (Fig. 2A, B). Interestingly, in Δ*hupA*Δ*hupS* strain 213 genes were affected at both analysed time points (while in the D *hupA* and Δ*hupS* it was only 43 and 64 genes, respectively) (Fig. S4). This may suggest that double deletion strain did not undergo a distinct transition between growth phases or that it was still at an earlier stage of growth than either the Δ*hupA* or Δ*hupS* strains.

Most often, the deletion of *hupA* and/or *hupS* resulted in transcriptional upregulation (88% and 55% of genes in the Δ*hupA* mutant, 80% and 71% in the Δ*hupS* and 83% and 76% in the Δ*hupA*Δ*hupS* strains during mid-log and early stationary phase, respectively) (Fig. 2A, S5B). To confirm the obtained results, ten representative genes from the putative *hupAS* regulon were chosen for replication analysis and their expression pattern was confirmed by an RT-PCR experiment (Pearson correlation coefficients 0.57 and 0.53 between Log_2_FC values for mid-log and early stationary phase, respectively) (Fig S5C).

To further compare the transcriptional changes between *hupA* and/or *hupS* deletion strains, we calculated the Pearson correlation coefficient of obtained Log_2_FC values from comparisons of mutant strains to the wild type strain (Fig. 2B). We found a moderate correlation between the *hupA* and *hupS* strains at both stages of growth (Pearson coefficient = 0.56 and 0.58 at mid-log and early stationary phase, respectively). Interestingly, the transcriptional changes detected in the double deletion mutant Δ*hupA*Δ*hupS* were similar to those in the single deletion strains only during mid-log phase (Pearson coefficient: 0.63 and 0.71 when compared to *hupA* and *hupS* strains, respectively), while during the early stationary phase this strain was visibly distinct (Pearson coefficient: 0.15 and 019 when compared to *hupA* and *hupS* strains, respectively). The high similarity between RNA-seq results obtained for all deletion mutants at an earlier stage of growth and the remarkable difference of the Δ*hupA*Δ*hupS* strain from the other two strains in early stationary phase could also be seen on the Principal Component Analysis (PCA) plot (Fig. S4, S5A).

Based on the pattern of expression differentially expressed genes could be divided into four major categories. The first group (HupA|S regulon) contained genes for which the presence of one HU homolog was sufficient to maintain the expression pattern. These genes were thus differentially expressed only in the Δ*hupA*Δ*hupS* strain – 148 and 255 genes during mid-log and early stationary phase, respectively. The second group (HupA&S regulon) required both HupA and HupS to maintain wild type levels of expression. Therefore, this group was comprised of genes whose expression changed in all tested strains, 149 and 11 genes during mid-log and early stationary phase, respectively. The last two groups (HupA regulon and HupS regulon) contained genes whose expression changed in the Δ*hupS* strain, but not in the Δ*hupA* strain or in the Δ*hupA* strain, but not in the Δ*hupS* strain. Surprisingly, the HupS regulon was larger than the HupA regulon during mid-log phase(257 and 98 genes, respectively) while the HupA regulon was larger during early stationary phase (Fig. 2C).

The larger number of genes under the control of HupA in the early stationary phase and under control of HupS during mid-log phase is somewhat contradictory to expectations based on the fact that HupA is the most abundant NAP during vegetative growth, while HupS levels increase during sporulation. The moderate overlap between the HupA and HupS regulons and the existence of HupA|S and HupA&S regulons, indicates some extent of cooperation between these two HU homologs. This cooperation may explain their synergistic impact on phenotype, although the details of such cooperation remain to be elucidated. On the other hand, the separate HupA and HupS regulons may correspond to the different binding pattern of each proteins. While HupA was shown to predominantly bind within the central region of the *S. coelicolor* chromosome, HupS in *S. venezuelae* preferentially bound within the arms regions [29, 32]. However, it should be highlighted that the identified regulons of HupA and HupS include those genes that are directly and indirectly regulated. Finally, it is worth highlighting that both HU homologs seemingly most often played the role of transcriptional repressors. A similar function has been noted for other NAPs such as Lsr2 in *S. venezuelae*, where removal of Lsr2 activated a number of secondary metabolite clusters [41]. In *S. coelicolor*, a novel NAP called Gbn was also found to have a suppressive effect on gene expression [38]. The comparable roles of HupA and HupS positions them among other proteins crucial for controlling *Streptomyces* metabolism.

### Deletion of *hupA* and *hupS* genes alters the expression of genes involved in chromosome structure and topology maintenance

**Fig. 3:**
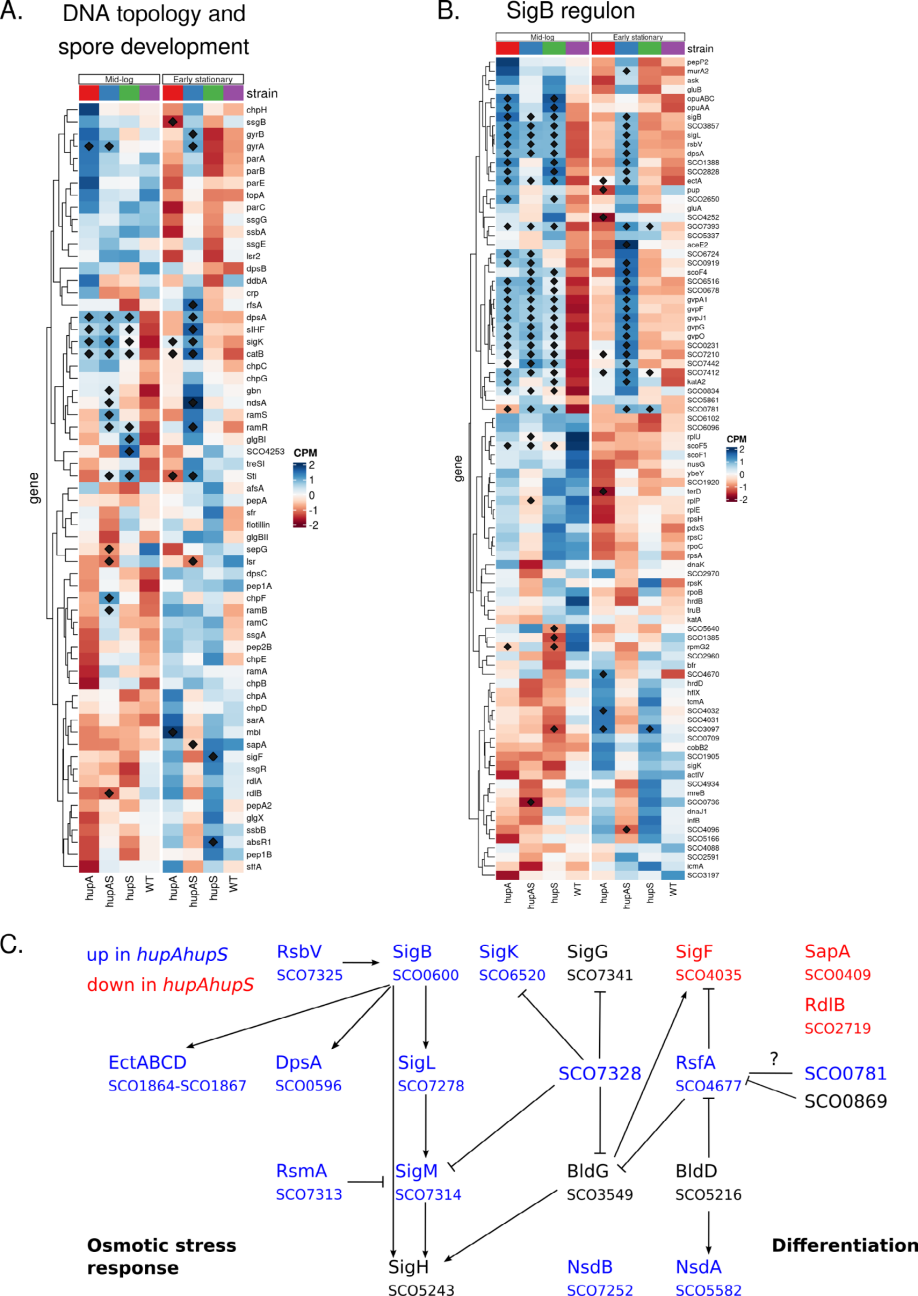
Deletion of *hupA* and/or *hupS* changes expression DNA topology maintenance and global regulators genes **(A)** Heatmap showing normalized expression (CPM) of genes connected with DNA topology and spore development in wild type, Δ*hupA*, Δ*hupS* and Δ*hupA*Δ*hupS s* trains during mid-log and early stationary phase. **(B)** Heatmap showing normalized expression (CPM) of SigB regulon genes in wild type, Δ*hupA*, Δ*hupS* and Δ*hupA*Δ*hupS s* trains during mid-log and early stationary phase. **(C)** Selected genes from differentiation and stress response networks that are upregulated (blue) or downregulated (red) in the Δ*hupA*Δ*hupS* strain during early stationary phase. **A**,**B** Genes marked with diamond were found to be significantly changed in comparison to the wild type strain (FDR ≤ 0.05)

The fragility of the Δ*hupA*Δ*hupS* strain’s spores, and to a lesser extent of the Δ*hupA* and Δ*hupS* strains, could be attributed either to lack of DNA protection by HU homologs or to transcriptional changes of genes vital for *S. coelicolor* spore maturation. Diminished resistance to stress conditions such as UV light, heat or free radicals has often been described for HU mutant strains of various bacterial species [33, 57–60]. Therefore we investigated how the transcriptional activity of genes important for DNA structure and topology or spore maturation was changed in *hupA* and/or *hupS* deletion mutants (Fig. 3A).

Since HupA is one of the most abundant NAPs in *S. coelicolor* during vegetative growth [30, 36], its deletion should be compensated by an upregulation of gene(s) encoding other NAP(s). Studies of *S. lividans* suggested that increased expression of *hupS* could partially suppress the effects of *hupA* deletion [37]. However, we have not found any evidence of *hupS* upregulation in the Δ*hupA* strain or *hupA* upregulation in the Δ*hupS* strain. Instead, in both the Δ*hupS* and Δ*hupA* mutants, we observed a significant increase in *dpsA* expression (*sco0596*), which encodes a NAP involved mainly in DNA protection in stressful conditions [40] and *sIHF* (*sco1480*), a NAP responsible for chromosome condensation and segregation [61, 62]. Expression of *dpsA* and *sIHF* increased during mid-log phase in all mutant strains as compared to the wild type (Log_2_FC for *dpsA* in Δ*hupA*: 3.45, in Δ*hupS*: 3.40 and in Δ*hupA*Δ*hupS*: 3.41; for *sIHF* in Δ*hupA*: 1.55, in Δ*hupS*: 1.24, in Δ*hupA*Δ*hupS*: 1.70), while in early stationary phase expression of those genes increased only in the Δ*hupA*Δ*hupS* strain (Log_2_FC for *dpsA*: 2.61; for *sIHF*: 1.56) (Fig. 3A, C). Deletion of *sIHF* was earlier shown to result in a phenotype similar to that of *hupA* and/or *hupS* mutants, namely reduced viability of spores and inhibition of sporulation [39]. This suggests an existence of a functional overlap between HupA, HupS and sIHF in *S. coelicolor.* Interestingly, transcription of a gene encoding another NAP named Gbn (*sco1839*) also increased in the Δ*hupA*Δ*hupS* strain (Log_2_FC Δ*hupA*Δ*hupS*: 1.75 and 1.31 during mid-log and early stationary phase, respectively) (Fig. 3A). Deletion of *gbn* had a different effect than *hupAhupS* deletion and led to accelerated development, while overexpression of *gbn* delayed sporulation [38]. Finally, unlike the above described NAP genes, the expression of *lsrL* (*sco4076*) decreased in the Δ*hupA*Δ*hupS* strain (Log_2_FC Δ*hupA*Δ*hupS* mid-log: -1.24, early stationary: -1.48). LsrL is a homolog of Lsr2, but little is known about its function in *Streptomyces*. Interestingly, the expression of *lsr2* remained unchanged in all tested strains.

The HupA protein is also crucial for DNA supercoiling homeostasis in *S. coelicolor* and cooperates with topoisomerase I (TopA) in maintaining chromosome topology [32]. Here, we did not detect any changes in expression of either *topA* (*sco3543*) or *parE/C* (*sco5822*, *sco5836*) genes encoding topoisomerase IV, but in strains with *hupA* deletion (*ΔhupA* and *ΔhupAΔhupS*), we observed an upregulation of the *gyrA/gyrB* operon (*sco3873-sco3874*) encoding gyrase, placing *gyrAB* in the HupA regulon (Fig. 3A). This result corroborates an earlier observation that the Δ*hupA* mutant was more sensitive to gyrase inhibition by novobiocin than the wild type strain [32]. Thus, the increased gyrase activity could be necessary to compensate for the lack of HupA.

Diminished UV and oxidative stress resistance of *hupA* and *hupS* spores could be linked to the role of HU in homologous recombination or RecA-dependant DNA repair [59, 63]. However, *recA* expression in *S. coelicolor hupA* and/or *hupS* mutants was not affected. Nevertheless, we found that some spore associated genes which were repressed in the Δ*hupA*Δ*hupS* strain during early stationary phase, e.g., *sapA* (*sco0409*) and *rdlB* (*sco2719*), were encoding spore coat proteins important for spore hydrophobicity [64–66] (Fig. 3A, C). These changes could account for increased spore sensitivity to stress factors.

Summarizing, genes encoding numerous proteins involved in DNA structure maintenance and protection, such as DpsA, sIHF and gyrase, were affected by either *hupA* or *hupS* deletion. This indicates at least partially independent functions of HupA and HupS in maintaining chromosome structure. Remarkably, in the mid-log phase, the elimination of either HupA or HupS led to upregulation of expression, thus placing those genes in the HupA&S regulon. This suggests that in this phase of growth, in the absence of HupA and HupS, other NAPs may compensate for their loss and maintain chromosome organisation. However, in the early stationary phase, the elimination of both HU homologs was required to activate other NAP encoding genes. This observation may be explained by modified chromosome structure during early stationary phase [28]. This may also explain the remarkably increased sensitivity of Δ*hupA*Δ*hupS* spores.

### HupA and HupS are a part of the *Streptomyces* regulatory network

The observed HupA- and HupS-dependent changes in global transcriptional activity might be explained by a direct impact of these NAPs on particular gene expression or by an indirect effect mediated by modified levels of regulators and/or sigma factors. To explore the latter possibility, we utilized the RNA-seq dataset to identify transcription regulators whose expression was altered in *hupA/hupS* deletion strains and whose regulatory networks have already been established. We found that genes encoding SigB (*sco0600*), ArgR (*sco1576*) and OsdR (*sco0204*) fulfilled these criteria [67–69]. Next, we analysed how HupA- and/or HupS-dependent modification of these genes impacted their regulatory networks. We also investigated whether HupA and HupS are involved in the regulatory network controlling the *S. coelicolor* life cycle.

SigB (*sco0600*) is a sigma factor, which acts as a major osmotic stress regulator and, through SigM (*sco7314*) and SigL (*sco7278*), regulates *Streptomyces* differentiation and stress response [67] (Fig. 3B, C). The expression of *sigB* increased in Δ*hupA* and Δ*hupS* strains during mid-log phase and in the Δ*hupA*Δ*hupS* strain during mid-log and early stationary phase (Fig. 3B, C, S5 B, C), placing *sigB* in the HupA&S regulon during mid-log phase and in the HupA|S regulon during early stationary phase. In *Streptomyces*, SigB activity is controlled by its anti-sigma factor RsbA (*sco0599*) and two anti-anti sigma factors: RsbB (*sco0598*) and RsbV (*sco7325*). Only *rsbV* expression was elevated in Δ*hupA* and/or Δ*hupS* strains. Out of 92 genes reported to belong to the SigB regulon and induced by KCl treatment [67] about one-fourth were upregulated in the Δ*hupA*Δ*hupS* strain at both time points (25 genes, hypergeometric test p-value: 6.65*10^–12^) (Fig. 3B). Remarkably, during mid-log phase, most of these genes were also upregulated in either Δ*hupA* or Δ*hupS* deletion strains, while during early stationary phase their expression was unchanged, reflecting the pattern of *sigB* expression levels. Genes from the SigB regulon that were upregulated by *hupA* and *hupS* deletions included *dpsA*, *sigL*, *sigM*, *ectABCD*, *rsbV* and *sco7590* (catalase) (Fig. 3B, C). Thus, the SigB network can serve as an example of HupA and HupS indirect influence, where changes of expression of a single sigma factor propagated through an entire regulatory network. However, a large fraction of genes from the SigB regulon were not found to be upregulated in either *hupA* nor *hupS* deletion strains. This could be explained by either the low sensitivity of RNA-seq method to small changes in gene expression or by the influence of other factors independent of *hupA* and/or *hupS* deletion.

Another global regulator that was affected by the double deletion of *hupA* and *hupS* genes was ArgR. The expression of *argR* (*sco1576*) increased during early stationary phase in the Δ*hupA*Δ*hupS* strain (Log_2_FC Δ*hupAΔhupS*: 2.69), placing it in the hupA|S regulon. According to published data, ArgR controls the expression of around 1500 genes and usually acts as a repressor [69], but for our analysis, we only considered 90 genes whose expression was altered by *argR* deletion at all tested time points. Surprisingly, we found that the upregulation of *argR* in the Δ*hupA*Δ*hupS* strain was accompanied by upregulation of ~ 30 genes belonging to ArgR cluster. These changes were observed during mid-log phase of all analyzed mutant strains and in the Δ*hupA*Δ*hupS* strain during early stationary phase (hypergeometric test p-value: 1.15*10^–16^). (Fig S6 A). In *E. coli*, HU proteins act as co-repressors with the GalR protein and *hupAB* deletion leads to the destabilization of repression loops and expression of the *gal* operon [70, 71]. A similar mechanism could perhaps explain the observed upregulation of the *arg* operon despite the increased expression of the *argR* repressor gene found in the Δ*hupAΔhupS* strain.

In Δ*hupA*, *ΔhupS* and *ΔhupAΔhupS* mutants, we found a significant increase in the expression of genes encoding the two-component system OsdKR (s *co0203-sco0204*) which thus falls into the HupA&S regulon (mid-log phase, Log_2_FC Δ*hupA*: 1.44 and 2.65, *ΔhupS*: 1.28 and 2.07, Δ*hupAΔhupS*: 1.41 and 2.50). OsdR plays an important role in the control of stress and development related genes, and is an orthologue of *Mycobacterium tuberculosis* DevR protein [68, 72]. The core regulon of OsdKR system lies between genes *sco0167* and *sco0219.* These genes were shown to be activated by OsdR and involved in stress response, spore maturation and nitrogen metabolism in *S. coelicolor* [68]. In all tested *hupA* and/or *hupS* strains, the expression of the “core” OsdR/K genes increased during mid-log phase, but genes belonging to the OsdR regulon located in other parts of *S. coelicolor* chromosome were not affected (Fig. S6B). Interestingly, 17 genes belonging to the OsdR regulon were earlier shown to be influenced by TopA depletion [36], suggesting that this cluster could be controlled by DNA supercoiling. OsdR itself requires presence of SCO2127 protein for expression [73]. However we have not observed any changes of *sco2127* in our dataset. The increased expression of genes belonging to the *osdR* regulon during early stationary phase in all tested strains is in agreement with previously published transcriptomic data [31, 68].

Lastly, we observed an induction of the *rsfA* gene (*sco4677*) but only in the Δ*hupA*Δ*hupS* strain (early stationary phase, Log_2_FC: 2.27). This gene encodes an anti-sigma factor interacting with SigF. *rsfA* null mutants are characterized by faster development [74, 75]. Additionally, RsfA is able to negatively regulate BldG (*sco3579*) by phosphorylation, linking it to the *Streptomyces* sporulation regulatory network [76]. Interaction of RsfA with two anti-anti-sigma factors was described [75], but only one of them (*sco0781*) was induced in both strains lacking *hupS.* Increased amount of RsfA and subsequent inhibition of SigF could explain the spore fragility of *ΔhupAΔhupS* since *sigF* null mutants in *Streptomyces* are characterized by lessened resistance to detergent treatment [77, 78]. Moreover, another anti-sigma factor, *sco7328*, was upregulated in the Δ*hupA*Δ*hupS s* train. This protein similarly to RsfA phosporylates BldG, but also inhibits activity of SigM, SigG (*sco7341*) and SigK [79] (Fig. 3C). The expression pattern of *rsfA*, *sigF* and *sco7328* places them in the HupA|S regulon.

To sum up, the analysis of the SigB regulon represents an example of a predictable secondary impact of *hupA* and *hupS* deletion resulting from *sigB* upregulation. In contrast, the ArgR regulon indicates the involvement of HU homologs in more complex regulatory circuits, while OsdR regulon expression could be influenced by structural changes of the *S. coelicolor* chromosome caused by either *hupA* or *hupS* deletion. On the other hand, RsfA upregulation could partially explain the slower growth of the Δ*hupAΔhupS* strain.

### Deletion of *hupA* and/or *hupS* affects production of secondary metabolites

**Fig. 4:**
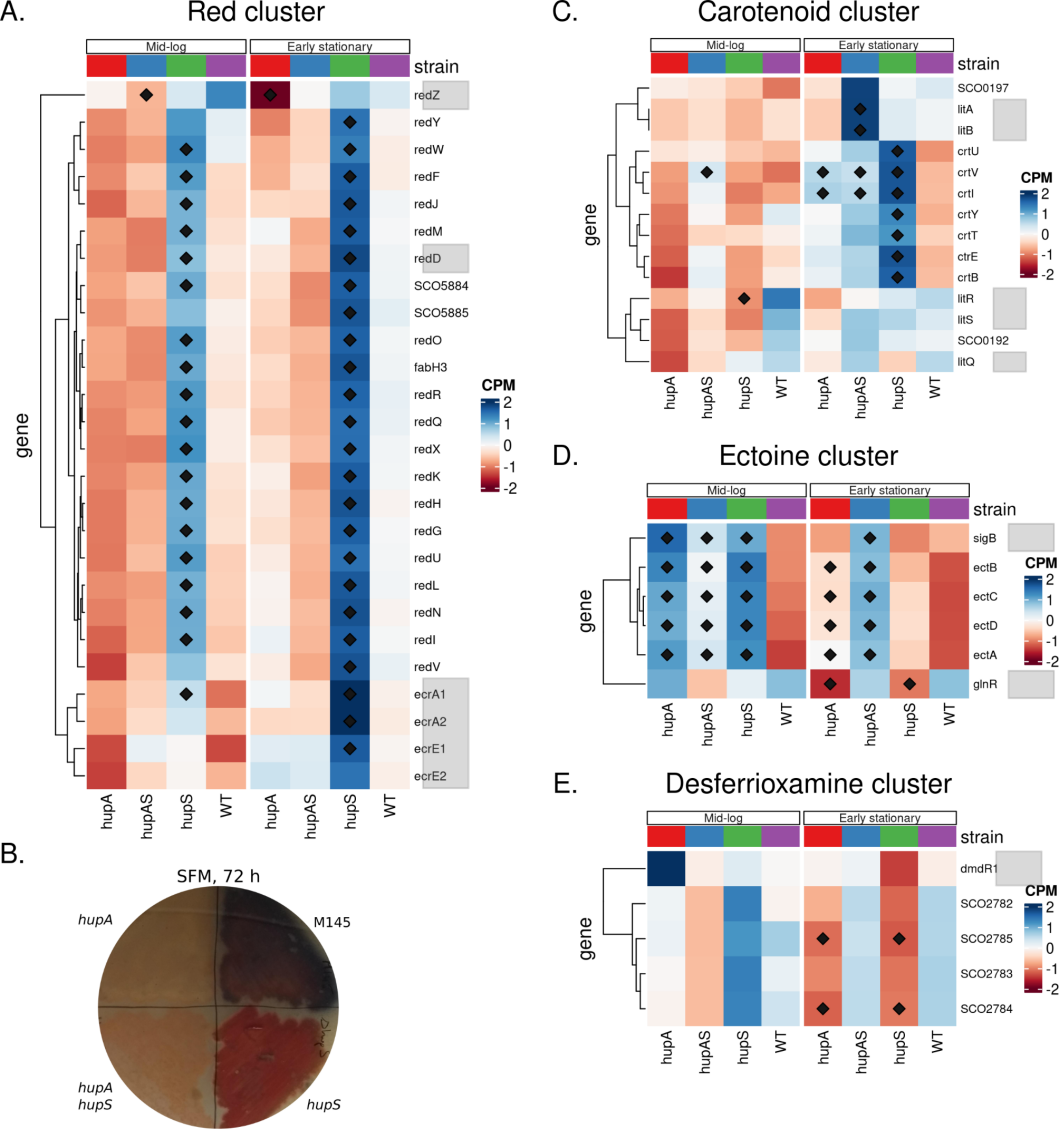
Deletion of *hupA* and/or *hupS* changes expression of genes associated with production of secondary metabolites **(A)** Heatmap showing normalized expression (CPM) of the red cluster genes in wild type, Δ*hupA*, Δ*hupS* and Δ*hupAΔhupS s* trains during mid-log and early stationary phase. **(B)** Production of undecyloprodigiosin (red metabolite) during plate culture of wild type, Δ*hupA*, Δ*hupS* and Δ*hupA*Δ*hupS s* trains incubated of solid SFM medium for 72 h in 30 °C. **(C)** Heatmap showing normalized expression (CPM) of the carotenoid gene cluster in wild type, Δ*hupA*, Δ*hupS* and Δ*hupA*Δ*hupS* strains during mid-log and early stationary phase. **(D)** Heatmap showing normalized expression (CPM) of the ectoine gene cluster in wild type, Δ*hupA*, Δ*hupS* and Δ*hupA*Δ*hupS* strains during mid-log and early stationary phase. **(E)** Heatmap showing normalized expression (CPM) of the desferrioxamine cluster genes in wild type, Δ*hupA*, Δ*hupS* and Δ*hupA*Δ*hupS* strains during mid-log and early stationary phase. **A**,**C**,**D**,**E** Genes marked with diamond were found to be significantly changed in comparison to the wild type strain (FDR ≤ 0.05). Genes encoding established transcription regulators are marked in grey

Given that the chromosome of *S. coelicolor*, similarly to other *Streptomyces* species, encodes numerous biosynthetic gene clusters, we set out to examine if the deletion of *hupA* and/or *hupS* in *S. coelicolor*, like *lsr2* deletion in *S. venezuelae*, could activate those clusters. We found that out of 22 secondary metabolic clusters present in *S. coelicolor* (Bentley, 2002) 4 exhibited changes of gene expression in *hupA* and/or *hupS* deletion strains. The most striking example was the *red* cluster (*sco5877-sco5898*) encoding the red oligopyrrole prodiginine antibiotic - undecyloprodigiosin [80]. Undecyloprodigiosins were suggested to have antimalarial and anticancer properties [81], and in *S. coelicolor* they were implicated in controlled cell death during development [82]. Expression of the *red* cluster is under the positive control of pathway specific regulators RedD and RedZ and is upregulated during stationary phase, especially during growth in liquid media [31, 83, 84]. The expression of almost the entire *red* cluster was elevated in the Δ*hupS* strain (but not in the D *hupA* or D *hup A* D *hupS* strains, placing it in the HupS regulon) at both tested time-points as compared to the wild type (Log_2_FC range for the *red* cluster, Δ*hupS* early stationary phase: 1.95–4.23), except for the transcription regulator *redZ* (*sco5881*). The fact that the double deletion of *hupA* and *hupS* did not lead to activation of the *red* cluster suggests that HupA presence is required for its expression. Indeed, *redZ w* as downregulated in the Δ*hupA* strain during stationary phase (Fig. 4A). Additionally, the two-component systems: *ecrA1/A2* (*sco2517-sco2518*) and *ecrE1/E2* (*sco6421-sco6422*) which are involved in transcriptional control of the *red* cluster [85, 86] were also upregulated during early stationary phase in Δ*hupS* strain. Notably, the overexpression of the *red* cluster was earlier observed in the strain with deletion of *sIHF* [39, 62]. Overproduction of RED antibiotic was confirmed by plate cultures showing an abundance of red pigment produced by the Δ*hupS* strain (Fig. 4B). Interestingly during growth on solid medium, Δ*hupA* and Δ*hupA*Δ*hupS* strains did not produce the characteristic blue pigment (actinorhodin) usually seen from *S. coelicolor* (Fig. 4B), which could be explained either by the growth delay or transcriptional influence of *hupA* deletion on the actinorhodin cluster expression. However our RNA-seq data did not show any significant changes in expression of the *act* cluster genes (Fig. S7). To sum up, HupA is required for the activation of gene encoding the activator RedZ while HupS inhibits expression of the *red* cluster genes possibly by downregulation of the two component system genes.

The other biosynthetic gene cluster affected by the elimination of HupS was the carotenoid cluster (*sco0185-sco0194*). Expression of the *crt* cluster was shown to be highest during mid-log phase [31]. The function of carotenoids in *Streptomyces* has not been fully established yet, but it is suggested that they are involved in protection from photo-oxidative damage [87]. Expression of the *crt* cluster was upregulated strongly in the Δ*hupS* strain during early stationary phase (Log_2_FC range for *crt* cluster = 2.09–4.54) and to a lesser degree in the Δ*hupA* (Log_2_FC = -0.05–2.72) and Δ*hupA*Δ*hupS* (Log_2_FC = 0.42–2.24) strains (Fig. 4C), placing it in the HupA&S regulon. Interestingly, genes belonging to the *crt* cluster are located between genes belonging to the *osdR* regulon, which expression was also elevated in either Δ*hupA* or Δ*hupS* mutants during mid-log phase (Fig S6). Expression of the *crt* cluster is light induced and controlled by *litQR (sco0193-sco0194)* and *litSAB* genes, with *litS* being essential for *crt* expression *(sco0195-sco0197*) (Takano, 2005). Notably, the expression of *litSAB* genes in the Δ*hupA* and/or Δ*hupS* strains was not significantly different from the wild type strain with the exception of *litA* and *litB*, which were upregulated in Δ*hupA*Δ*hupS* strain during early stationary phase (Fig. 4C).

Elimination of HupA and/or HupS activated the ectoine cluster (*sco1864-sco1887)*. Ectoine production in *Streptomyces* has been linked with survival in high salt or temperature conditions [88]. Earlier transcriptional studies showed that *ect* cluster expression is highest during mid-log phase and decreases at later stages of growth on both solid and liquid media [31, 83, 89]. Deletion of *hupA* and/or *hupS* genes led to an overexpression of *ectABCD* genes at both analysed time points (Log_2_FC range for *ect* cluster mid-log phase, *ΔhupA*: 2.54–2.94; *ΔhupS*: 2.68–4.00; *ΔhupAΔhupS*: 1.37–2.15) thus placing this cluster in the HupA&S regulon (Fig. 4D). The ectoine cluster was shown to be negatively controlled by the GlnR transcription factor (*sco4159*) [90] and expression of *glnR* decreased in the Δ*hupA* and Δ*hupS* strains during early stationary phase. Additionally *ect* cluster expression is dependent upon SigB [67, 91], and expression of *sigB* was elevated in all three *hupA* and/or *hupS* deletion strains (Fig. 4D). Thus, ectoine cluster activation may be explained by increased levels of SigB and lowered levels of GlnR in the absence of HU homologs.

Contrary to the previously described clusters, the desferrioxamine cluster *(sco2782-sco2785)* was downregulated in the absence of HupA or HupS. The *des* cluster encodes genes necessary for the production of desferroixamine, a nonpeptide hydroxamate siderophore [92]. Desferrioxamine are produced by many *Streptomyces* species in iron deficiency conditions and at an early stage of growth on solid media [89, 93]. Expression of *desABCD* genes decreased in the Δ*hupA* and Δ*hupS* strains during early stationary phase (Log_2_FC range Δ*hupA*: -2.24 to -3.08; Δ*hupS*: -2.65 to -3.30), but not in the double deletion Δ*hupA*Δ*hupS* strain (Fig. 4E). *desABCD* expression is governed by the iron repressor *dmdR1 (sco4394)* [94] but *dmdR1* transcription was not affected by *hupA* and/or *hupS* deletion (Fig. 4E). Thus, the mechanism of *des* cluster regulation by HupA and HupS is unclear.

To sum up, we found that expression of four BGCs was modified in the absence of HupA and/or HupS. Expression of secondary clusters responsible for the production of RED and carotenoid compounds were activated by *hupS* deletion, however the presence of HupA was required for this activation. That suggests an interplay between both HU homologs in the transcriptional regulation of these clusters. In contrast, the ectoine cluster was upregulated in the absence of at least one HU homolog. For three out of four clusters, the changes of expression could be at least partially explained by modified levels of the pathway specific (RedZ) or global (SigB) regulators.

### Effective response to osmotic stress depends upon the presence of HupA

Several sigma factors whose expression levels were affected by *hupA* and/or *hupS* deletion participate in *S. coelicolor’s* response to osmotic stress (i.e. SigB). Similarly, ectoine plays a crucial role in *Streptomyces* survival in high-salt environments and its biosynthetic gene cluster expression was elevated in *hupA* and/or *hupS* deletion mutants [88]. These observations prompted us to determine the effect of *hupA* and/or *hupS* deletion on *S. coelicolor* survival and gene expression in osmotic stress. To this end, we cultured the Δ*hupA*, Δ*hupS* and Δ*hupA*Δ*hupS* strains in liquid YEME/TSB medium supplemented with 0.5 M NaCl, to assess their growth rate, and next, to determine the transcription profile using RNA-seq (Table S3).

**Fig. 5:**
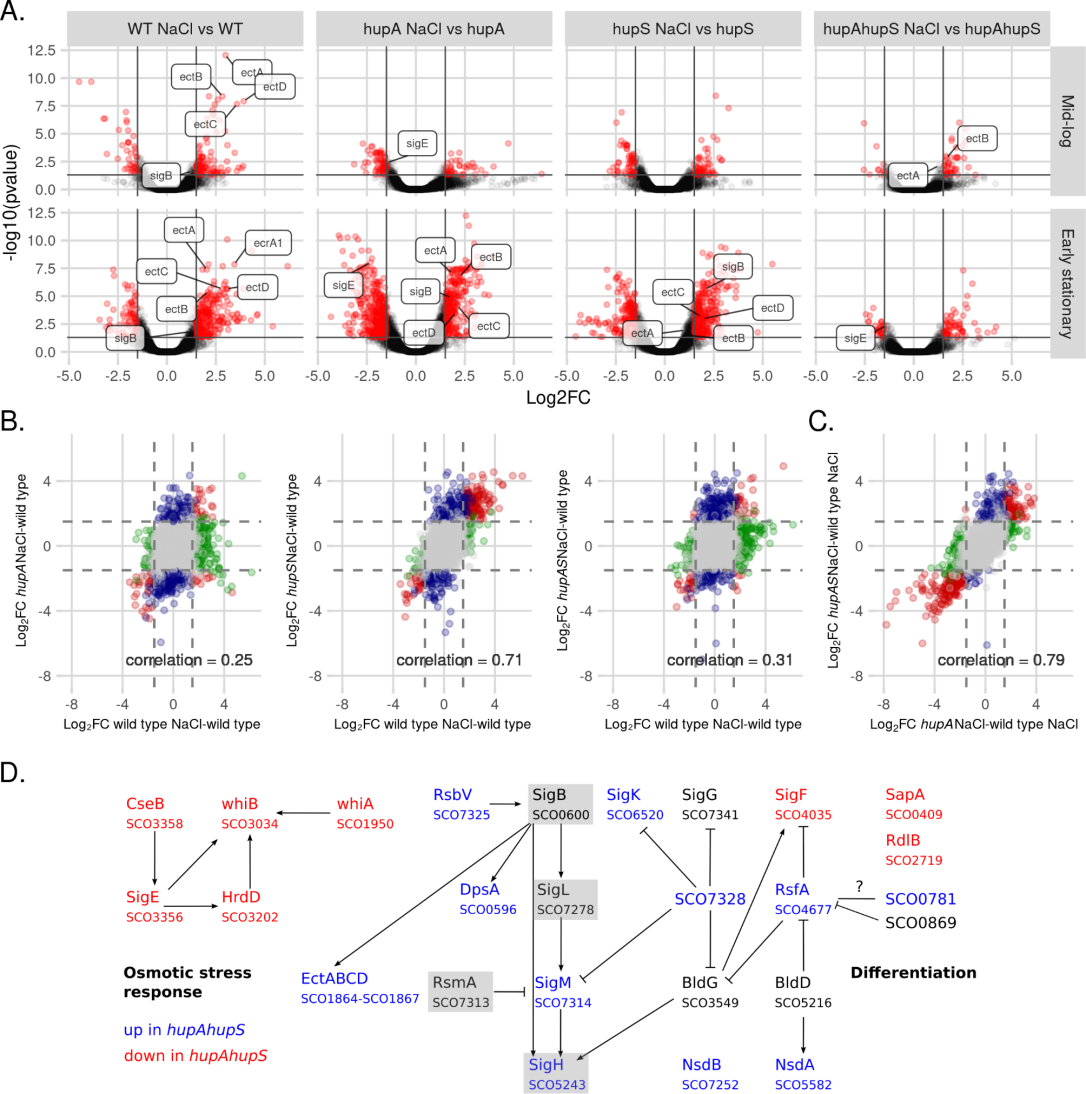
*hupA* deletion changes *S. coelicolor* response to growth in osmotic stress **(A)** Volcano plots showing changes in gene expression in wild type, Δ*hupA*, *ΔhupS* and Δ*hupAΔhupS* strains from mid-log and early stationary phase of growth in NaCl supplemented medium (0.5 M NaCl) compared to the same strain grown in standard YEME/TSB medium. For each strain significantly changed genes (FDR ≤ 0.05, |Log_2_FC| > 1.5) are shown in red. Chosen differentially expressed genes are labeled. **(B)** Scatterplots showing correlation between wild type and Δ*hupA*, *ΔhupS* and Δ*hupAΔhupS* Log_2_FC values during early stationary phase in medium supplemented with salt. Log_2_FC values were calculated from comparison with wild type strain grown in standard medium. Pearson correlation coefficient is shown on each plot. Genes found to be significant in both comparisons (FDR ≤ 0.05, |Log_2_FC| > 1.5) are shown in red, while genes significant in only one of the comparisons are marked as blue or green, grey dots represent genes not affected by *hupA* and/or *hupS* deletions. **(C)** Correlation between Δ*hupA* and Δ*hupAΔhupS* transcriptomes during early stationary phase in NaCl supplemented medium. The Pearson correlation coefficients are shown on the plot. Genes found to be significant in both comparisons (FDR ≤ 0.05, |Log_2_FC| > 1.5) are shown in red, while genes significant in only one of the comparisons are marked as blue or green, grey dots represent genes not affected by *hupA* and double *hupAhupS* deletions. **(D)** Regulatory networks involving selected upregulated (blue) or downregulated (red) genes in Δ*hupA*Δ*hupS* strain during early stationary phase in NaCl supplemented medium (0.5 M NaCl)

Addition of 0.5 M NaCl slowed down growth of all tested strains, but the inhibition of growth was most pronounced for the Δ*hupA* and Δ*hupA*Δ*hupS* strains (Fig. S3). NaCl supplementation altered expression of a substantial number of genes in the wild type strain, with more genes affected during the early stationary than the mid-log phase (142 and of 338 genes, respectively). Deletion of *hupA* or *hupS* led to significant changes in transcriptional activity, affecting 659 and 455 genes, respectively, during early stationary phase compared to strains cultured in normal medium. However, the double deletion mutant was largely unaffected by osmotic stress, with only 95 changed genes during early stationary phase in the NaCl-supplemented medium (Fig. 5A). Next, we calculated the correlations between individual strains’ responses to the osmotic stress. The Δ*hupS* strain transcriptome was the most similar to the wild type strain (Pearson coefficient = 0.71), while the Δ*hupA* and *ΔhupAΔhupS* strains were significantly different from the wild type strain (Pearson coefficient 0.25 and 0.31, respectively). Both strains lacking the *hupA* gene were remarkably similar to each other with a 0.79 correlation coefficient and 432 shared genes (Fig. 5B, C, S8).

These observations suggest that the presence of HupA is necessary for the *S. coelicolor* response to osmotic stress. This situation could be partly explained by the fact that several genes overexpressed in the wild type strain in NaCl supplemented medium, like *ectABCD* or *sigB*, are a part of the HupA&S regulon (Fig. 5A, D). SigB was not upregulated in Δ*hupA*Δ*hupS* strain during osmotic stress when compared to wild type strain also grown in NaCl supplemented medium. Moreover, among the genes most affected by *hupA* deletion, we found *sigE (sco3356);* a sigma factor related to osmotic stress [95, 96]. SigE controls the expression of genes mainly involved in maintaining cell wall and membrane integrity [96]. HupA could be one of SigE’s binding partners [97]. Lack of SigE could explain some of the transcriptomic changes observed in both Δ*hupA* strains. Indeed, analysis of the SigE regulon revealed the presence of 42 genes that were strongly repressed in either the Δ*hupA* or *ΔhupAΔhupS* strains but induced in the wild type and Δ*hupS* strains during osmotic stress (hypergeometric test p-value: 2.49 * 10^–14^) (Fig. 5D, S8 D). On the other hand, in vitro studies concerning the HupA protein showed that its binding to DNA is affected by NaCl concentration [10]. Perhaps altered distribution of HupA on the *S. coelicolor* chromosome is one of the sources of observed transcriptional alterations.

### Co-expressed gene clusters within HupAS regulons

**Fig. 6:**
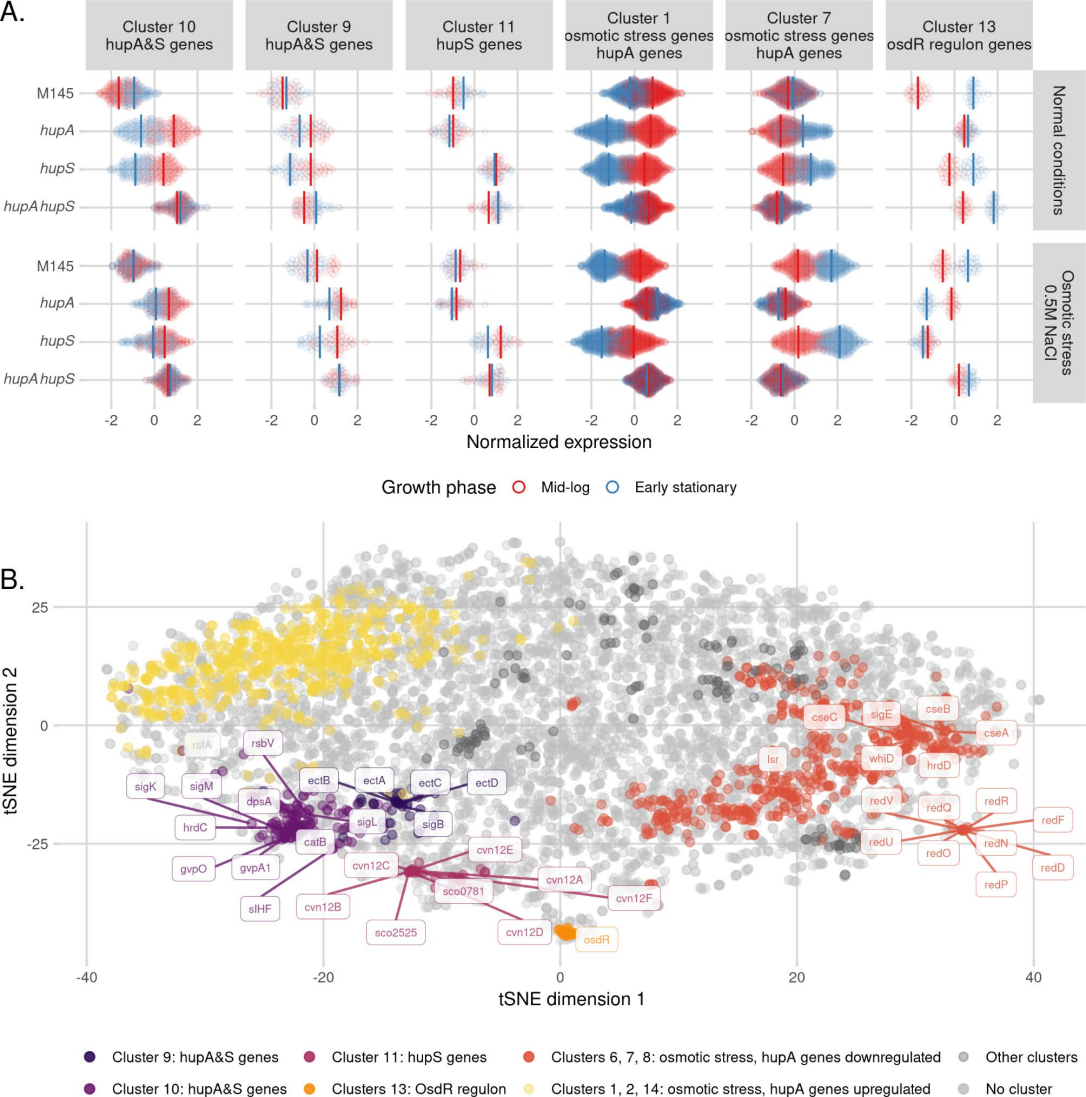
Co-expressed clusters of genes within *hupAS* regulons **(A)** Co-expressed clusters identification - results of clust analysis of wild type, Δ*hupA*, Δ*hupS* and Δ*hupA*Δ*hupS* transcriptomes from mid-log (red) and early stationary (blue) phase of growth in normal and NaCl supplemented medium. Plot shows six chosen clusters out of 15 obtained. **(B)** t-distributed stochastic neighbour embedding (t-SNE) analysis of normalized wild type Δ*hupA*, Δ*hupS* and Δ*hupA*Δ*hupS* transcriptomes from mid-log and early stationary phase of growth in normal and NaCl supplemented medium. HupA&S regulon (cluster 9 – dark purple, cluster 10 – purple, cluster 13 - orange), HupS regulon (cluster 11 – pink), HupA regulon clusters affected by growth in NaCl supplemented medium (clusters 6, 7, 8 – red, clusters 1, 2, 14 – yellow). Positions of genes associated with stress response, secondary metabolite production, spore development and NAPs are shown on the plot

Lastly, to improve gene classification into the four major regulons (HupA, HupS, HupA&S and HupA|S) by taking into account the influence of salt, we performed a cluster analysis of the entire dataset obtained for the four strains tested at two timepoints under two growth conditions. Using the *clust* programme we found 15 co-expressed clusters of genes (with sizes ranging between 11 and 577) (Fig. S9), containing a total of 1601 *S. coelicolor* genes. Among the obtained co-expressed clusters, three contained genes whose expression seemed to be affected by the lack of either of the HU homologs (clusters 9, 10 and 13) and thus belonging to the HupA&S regulon. Two of those co-expressed clusters, 9 (39 genes) and 10 (142 genes), included many genes identified in the earlier differential expression analysis as controlled by both HupA and HupS, such as: *dpsA*, *sIHF*, *sigM*, *sigL*, *rsbV*, *catB*, *sigK* in cluster 10, and: *sigB*, *ectABCD* in cluster 9. The expression of genes from cluster 10 seemed to be largely unaffected by growth in osmotic stress, while genes belonging to cluster 9 were upregulated during early stationary phase in osmotic stress conditions in all tested strains (Fig. 6). Interestingly, cluster 13 contained only genes located between 150 kb and 213 kb on the *S. coelicolor* genome and belonging to the OsdR regulon described earlier. Possibly, the control of cluster 13 expression involves changes in chromosome organization and/or supercoiling, explaining its sensitivity to both *hupA* and/or *hupS* deletion and TopA depletion [36] (Fig. 6).

Cluster 11 (33 genes) contained genes whose expression changed only in Δ*hupS* or Δ*hupA*Δ*hupS* strains, thus constituting the HupS regulon. The expression of those genes was not influenced by growth in osmotic stress conditions and was also similar at both tested growth stages. Seemingly, those genes were controlled solely by HupS. This group included the *cvn12* conservon (*sco2879-sco2884*), an anti-anti sigma factor *sco0781*, a putative LysR regulator (*sco2734*), a putative stress response protein (*sco5806*), and an operon *sco2521-sco2526.* SCO2525 is a putative methyltransferase necessary for normal growth of *S. coelicolor* [98] (Fig. 6). Genes affected only by *hupA* deletion (HupA regulon) belonged to six co-expressed clusters (Clusters 1, 2, 6, 7, 8, 14), and their expression changed only during osmotic stress. Cluster 7 contained, among others, genes belonging to the *red* cluster (Fig. S10), *sigE*, and *lsrL*. The expression of those genes increased during early stationary phase in osmotic stress in the wild type and *ΔhupS* strain, but remained low in the Δ*hupA* and *ΔhupAΔhupS* strains (Fig. 6, S9). This analysis confirmed that genes belonging to HupA and HupA&S regulons are involved in the osmotic stress response, while HupS regulon genes are not sensitive to increased salt concentration.

## Conclusions

**Fig. 7:**
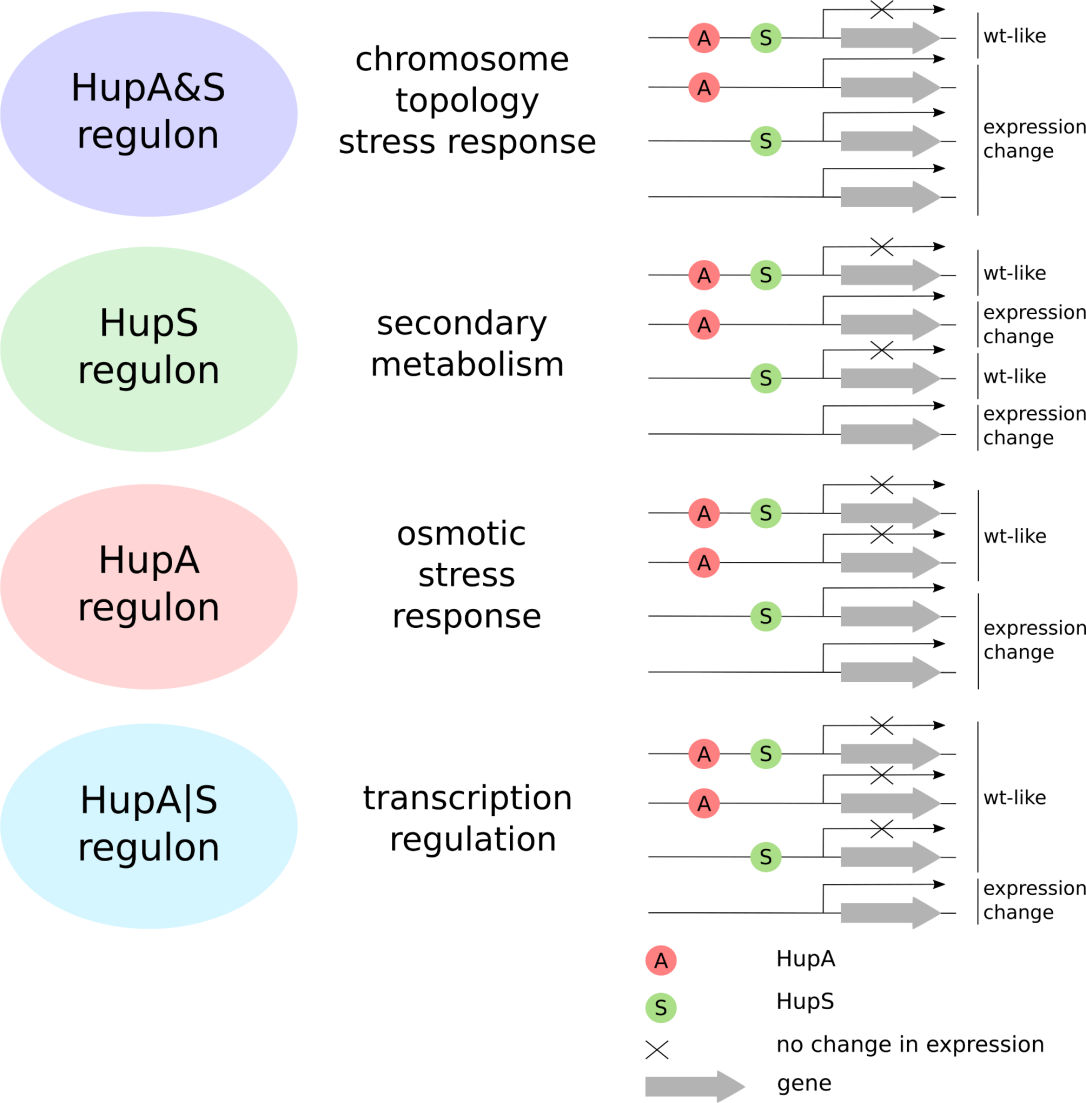
HupA and HupS regulons A scheme showing the criteria used to divide genes into specific groups: HupA regulon, HupS regulon, HupA&S regulon, and HupA/S regulon, based on the strain in which a change in expression pattern was observed compared to the wild-type strain

In summary, the presence of both HupA and HupS is necessary for proper growth and development of *S. coelicolor*. The absence of both HupA and HupS results in severe growth inhibition and impaired stress survival, significantly more pronounced than that of either single deletions strains, indicating a synergy between the actions of the two HU homologs. The increased sensitivity of spores to DNA damaging factors may be explained by diminished protection of DNA. RNA-seq analysis showed that by binding to DNA, HupA and HupS act as global transcription factors, altering the expression of multiple genes, mostly upregulating them. Genes upregulated in the absence of at least one of the HU homologs were involved in DNA protection (NAPs, gyrase), transcription regulation (e.g. sigma factors) or stress survival (e.g. osmotic stress). HupS was involved in controlling the expression of secondary metabolite clusters (e.g. the *red* cluster), while HupA’s control of gene expression, separate from HupS, was mostly evident during growth in osmotic stress (Fig. 7). The identification of HupA&S and HupA|S regulons suggests a cooperation between the two HU homologs in *Streptomyces.*

## Electronic supplementary material

Below is the link to the electronic supplementary material.

Supplementary Material 1: Fig. S1. Growth and sporulation of ΔhupAΔhupS strain. A. Growth curves of wild type (purple), ΔhupA (red), ΔhupAΔhupS (blue), ΔhupAΔhupS::hupA (orange) strains in liquid YEME/TSB medium. Bold lines represent logistic model while striped lines show calculated half time. B. Microscopic images of spores of wild type S. coelicolor and ΔhupAΔhupS strains from 7-day old colonies grown on SFM solid medium. Scale 5 μm. Fig. S2 Impact of hupA and/or hupS deletion on S. coelicolor growth in the presence of H2O2 A. Growth curves of wild type (purple), ΔhupA (red), ΔhupS (green) and ΔhupAΔhupS (blue) strains cultured in ‘79’ medium supplemented with increasing concentrations of H2O2. Solid lines show the growth curve smoothed by the loess algorithm. B. Growth of wild type, ΔhupA, ΔhupS and ΔhupAΔhupS strains on solid SFM medium containing increasing concentrations of H2O2. Pictures were taken after 5 days of incubation in 30°C. Fig. S3 Impact of osmotic stress on the growth of wild type, ΔhupA, ΔhupS and ΔhupAΔhupS strains Growth curves of wild type, ΔhupA, ΔhupS and ΔhupAΔhupS strains in 30 ml of standard (red) and NaCl supplemented (blue, 0.5 M NaCl) YEME/TSB medium. Vertical lines show growth half-time calculated by logistic model. Fig. S4 Comparison of transcriptomes of ΔhupA, ΔhupS, ΔhupAΔhupS strains during different phases of growth A. Scatterplots showing correlation between ΔhupA, ΔhupS and ΔhupAΔhupS transcriptomes from mid-log phase compared to early stationary phase. Pearson correlation coefficient is shown on each plot. Genes found to be significant in both comparisons (FDR ≤ 0.05, |Log2FC| > 1.5) are shown in red, while genes significant in only one of the comparisons are marked as blue or green, grey dots represent genes not affected by hupA and/or hupS deletions. B. Venn diagrams showing significantly changed genes common for analyzed strains ΔhupA, ΔhupS and ΔhupAΔhupS during mid-log and early stationary phase. Fig. S5 RNA-seq and RT-PCR analysis of gene expression A. Principal component analysis (PCA) of the normalized RNA-seq CPM (Counts Per Million) data of S. coelicolor strains in response to hupA (red), hupS (green) or hupAhupS (blue) deletion during mid-log (circles) or early stationary phase (triangles). B. Correlation between Log2FC values obtained from RNA-seq and RT-PCR for genes: dpsA, sigB, gyrB, fabH3, ecrA1, sIHF, lsr, sco0781, gvpA1 from ΔhupA (red), ΔhupS (green) and ΔhupAΔhupS (blue) strains when compared to the wild type strain. C. Table showing Log2FC values of several genes in strains ΔhupA, ΔhupS and ΔhupAΔhupS when compared to the wild type strain during mid-log and early stationary phase, NA value indicates that FDR value was above the 0.05 threshold. Fig. S6 Genes clusters regulated by HupA and HupS A. Heatmap showing normalized expression (CPM) of ArgR regulon genes for ΔhupA, ΔhupS and ΔhupAΔhupS strains during mid-log and early stationary phase. B. Heatmap showing normalized expression (CPM) of OsdR regulon genes for ΔhupA, ΔhupS and ΔhupAΔhupS strains during mid-log and early stationary phase. A, B Genes marked with diamond were found to be significantly changed in comparison to the wild type strain (FDR ≤ 0.05). Fig. S7 Heatmap showing normalized expression (CPM) of actinorhodin cluster genes for ΔhupA, ΔhupS and ΔhupAΔhupS strains during mid-log and early stationary phase. Genes marked with diamond are those whose expression was found to be significantly altered in comparison to the wild type strain (FDR ≤ 0.05). Fig. S8 Changes of gene expression induced by osmotic stress conditions A. Volcano plots showing modified gene expression in ΔhupA, ΔhupS and ΔhupAΔhupS during mid-log and early stationary phase in medium supplemented with 0.5 M NaCl compared to the wild type strain. For each strain significantly changes genes (FDR ≤ 0.05, |Log2FC| > 1.5) are shown in red. Chosen differentially expressed genes are labelled. B. Venn diagrams showing significantly changed genes common for strains ΔhupA, ΔhupS and ΔhupAΔhupS during mid-log or early stationary phase in medium supplemented with 0.5 M NaCl. C. Heatmap showing normalized expression (CPM) of 150 genes with the lowest FDR value calculated for ΔhupAΔhupS strain and wild type comparison in wild type, ΔhupA, ΔhupS and ΔhupAΔhupS strains during mid-log and early stationary phase in NaCl supplemented medium (0.5 M NaCl). D. Heatmap showing normalized expression (CPM) of SigE regulon genes for ΔhupA, ΔhupS and ΔhupAΔhupS strains during mid-log and early stationary phase in NaCl supplemented medium (0.5 M NaCl). C, D Genes marked with diamond were found to be significantly changed in comparison to the wild type strain (FDR ≤ 0.05, |Log2FC| > 1.5). Fig. S9 All co-expressed clusters of genes identified based on transcriptional changes in ΔhupA, ΔhupS and ΔhupAΔhupS Results of clust analysis of wild type, ΔhupA, ΔhupS and ΔhupAΔhupS transcriptomes from mid-log (red) and early stationary (blue) growth in normal and NaCl supplemented medium. Fig. S10 Modified expression of the genes within biosynthetic gene clusters, ΔhupA, ΔhupS and ΔhupAΔhupS strains during growth at osmotic stress as compared to the wild type strain A. Heatmap showing normalized expression (CPM) of the red cluster genes in wild type, ΔhupA, ΔhupS and ΔhupAΔhupS strains during mid-log and early stationary phase in NaCl supplemented medium (0.5 M NaCl). B. Heatmap showing normalized expression (CPM) of the carotenoid cluster genes in wild type, ΔhupA, ΔhupS and ΔhupAΔhupS strains during mid-log and early stationary phase in NaCl supplemented medium (0.5 M NaCl) C. Heatmap showing normalized expression (CPM) of the ectoine cluster genes in wild type, ΔhupA, ΔhupS and ΔhupAΔhupS strains during mid-log and early stationary phase in NaCl supplemented medium (0.5 M NaCl) D. Heatmap showing normalized expression (CPM) of the desferrioxamine cluster genes in wild type, ΔhupA, ΔhupS and ΔhupAΔhupS strains during mid-log and early stationary phase in NaCl supplemented medium (0.5 M NaCl) A, B, C, D Genes marked with diamond were found to be significantly changed in comparison to the wild type strain (FDR ≤ 0.05).

Supplementary Material 2

## Data Availability

The raw RNA-Seq data, as well as the processed data generated in this study, have been deposited in the ArrayExpress database (EMBL-EBI) under accession code E-MTAB-13846.
