# Supplementary material for: The role of two major nucleoid-associated proteins in Streptomyces, HupA and HupS, in stress survival and gene expression regulation
Source: Microb Cell Fact. 2024 Oct 14;23:275. doi: 10.1186/s12934-024-02549-0 (PMC11472566; doi:10.1186/s12934-024-02549-0)
Supplement: Supplementary file 1 — Supplementary Material 1: Fig. S1. Growth and sporulation of ΔhupAΔhupS strain. A. Growth curves of wild type (purple), ΔhupA (red), ΔhupAΔhupS (blue), ΔhupAΔhupS::hupA (orange) strains in liquid YEME/TSB medium. Bold lines represent logistic model while striped lines show calculated half time. B. Microscopic images of spores of wild type S. coelicolor and ΔhupAΔhupS strains from 7-day old colonies grown on SFM solid medium. Scale 5 μm. Fig. S2 Impact of hupA and/or hupS deletion on S. coelicolor growth in the presence of H2O2 A. Growth curves of wild type (purple), ΔhupA (red), ΔhupS (green) and ΔhupAΔhupS (blue) strains cultured in ‘79’ medium supplemented with increasing concentrations of H2O2. Solid lines show the growth curve smoothed by the loess algorithm. B. Growth of wild type, ΔhupA, ΔhupS and ΔhupAΔhupS strains on solid SFM medium containing increasing concentrations of H2O2. Pictures were taken after 5 days of incubation in 30°C. Fig. S3 Impact of osmotic stress on the growth of wild type, ΔhupA, ΔhupS and ΔhupAΔhupS strains Growth curves of wild type, ΔhupA, ΔhupS and ΔhupAΔhupS strains in 30 ml of standard (red) and NaCl supplemented (blue, 0.5 M NaCl) YEME/TSB medium. Vertical lines show growth half-time calculated by logistic model. Fig. S4 Comparison of transcriptomes of ΔhupA, ΔhupS, ΔhupAΔhupS strains during different phases of growth A. Scatterplots showing correlation between ΔhupA, ΔhupS and ΔhupAΔhupS transcriptomes from mid-log phase compared to early stationary phase. Pearson correlation coefficient is shown on each plot. Genes found to be significant in both comparisons (FDR ≤ 0.05, |Log2FC| > 1.5) are shown in red, while genes significant in only one of the comparisons are marked as blue or green, grey dots represent genes not affected by hupA and/or hupS deletions. B. Venn diagrams showing significantly changed genes common for analyzed strains ΔhupA, ΔhupS and ΔhupAΔhupS during mid-log and early stationary phase. Fig. S5 RNA-seq and RT-P [file 12934_2024_2549_MOESM1_ESM.pdf]

Supplementary Data

Table S1

Streptomyces coelicolor strains used in the study

Strain	Relevant genotype	Sources or reference
M145	SCP1- SCP2-	Bentley et al., 2002
ASMK011	M145 *ΔhupA::scar*	Strzałka et. al., 2022
K304	M145 *ΔhupS::higro*	Salerno et. al., 2009
ASMK019	M145 *ΔhupA::scar ΔhupS::higro*	This study
ASMK019.2	M145 *ΔhupA::scar ΔhupS::higro* pSET152*hupA*	This study

Table S2

Oligonucleotides used in the study

Primer name	Sequence
sigma_FW	GGCTGGAGGCAAGGTTCGTG
sigma_RV	GCCGAGGCGATGTCGATCTC
gvp_FW	CGACGTGCTGGAGCTTGTTCT
gvp_RV	CAGGATCTCGATGCCGACCA
dpsA_FW	GCACCTGACCCTCAAGCAC
dpsA_RV	GTCGTCCCACTTCCGCTC
ecrA_FW	GGCACCCGCAACGACACC
ecrA_RV	CCAGCAGCGTCCAGCCGA

fab_FW	TGGAGCGGACGATGGACAC
fab_RV	GCCAGGTCGGAGGTGAGG
lsr_FW	CGTGTCGTGGTCACTCTCTTTG
lsr_RV	GGCATTGGCTTCGTTCAGGT
nsdA_FW	GCCCACCGCCCAGAAGTA
nsdA_RV	GCTGGAGAGGATGTAGGAGCC
sigB_FW	CGACGACTTCCACGCCCT
sigB_RV	ATGTCCTTCTGGGTGAGTTCCT
rfsA_FW	TTCCGGCTCACCCTGTACG
rfsA_RV	TCGACCAGCAGCAGACCC
hrdBRT_f	TGCTCTTCCTGGACCTCATC
hrdBRT_r	GTAGCCCTTGGTGTAGTCGAA
hupA_pSET_FW	GCTTGGGCTGCAGGTCGACTCGAACTACATCATCCCCAG
hupA_pSET_RV	CGATATCGCGCGCGGCCGCGCTACTTGCCCTTGGCGGCTTC

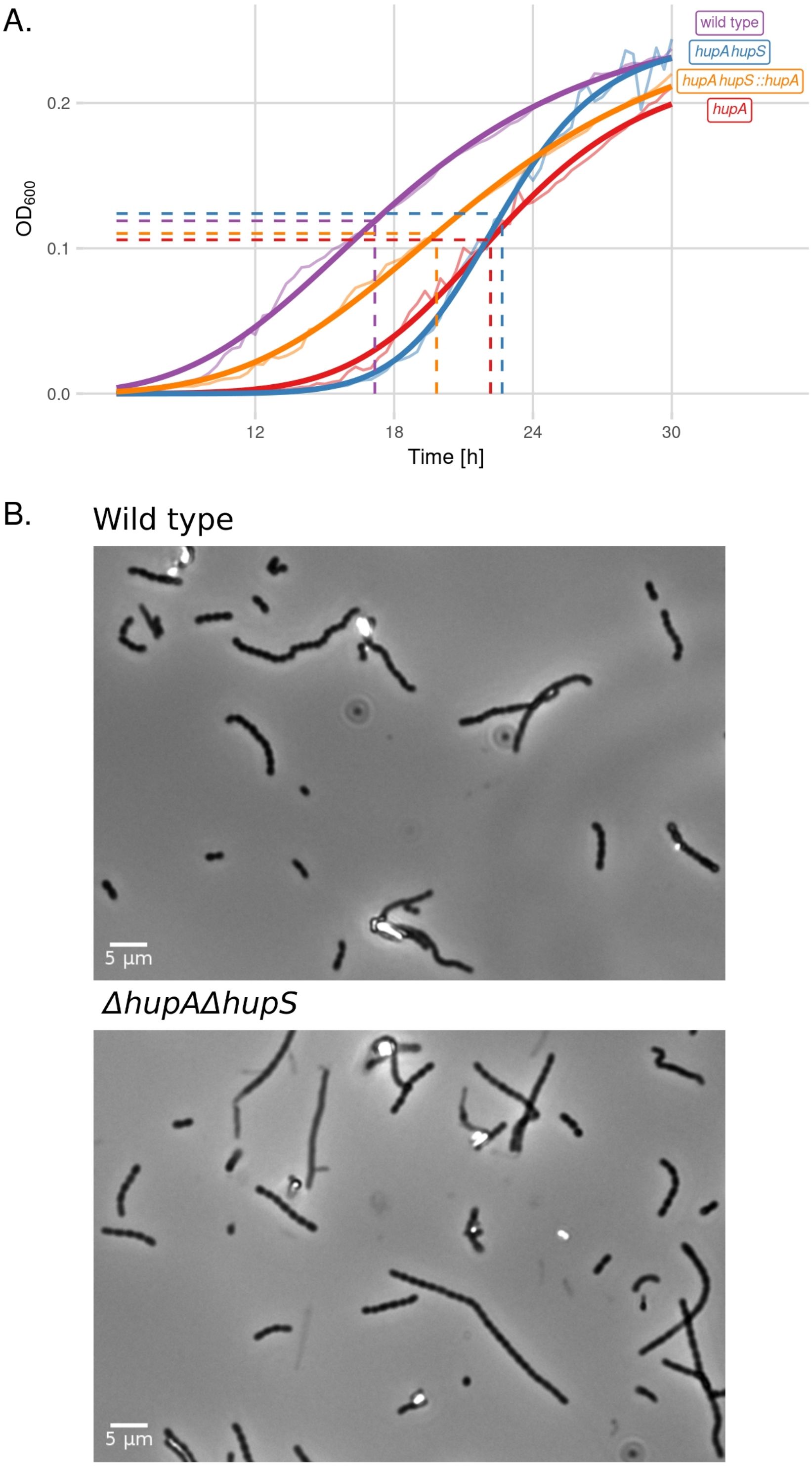
A.
wild type
hupA hupS
hupA hupS ::hupA
hupA
0.2
0.1
0.0
OD600
12
18
24
30
Time [h]
B.
Wild type
5 µm
ΔhupAΔhupS
5 µm

Figure S1

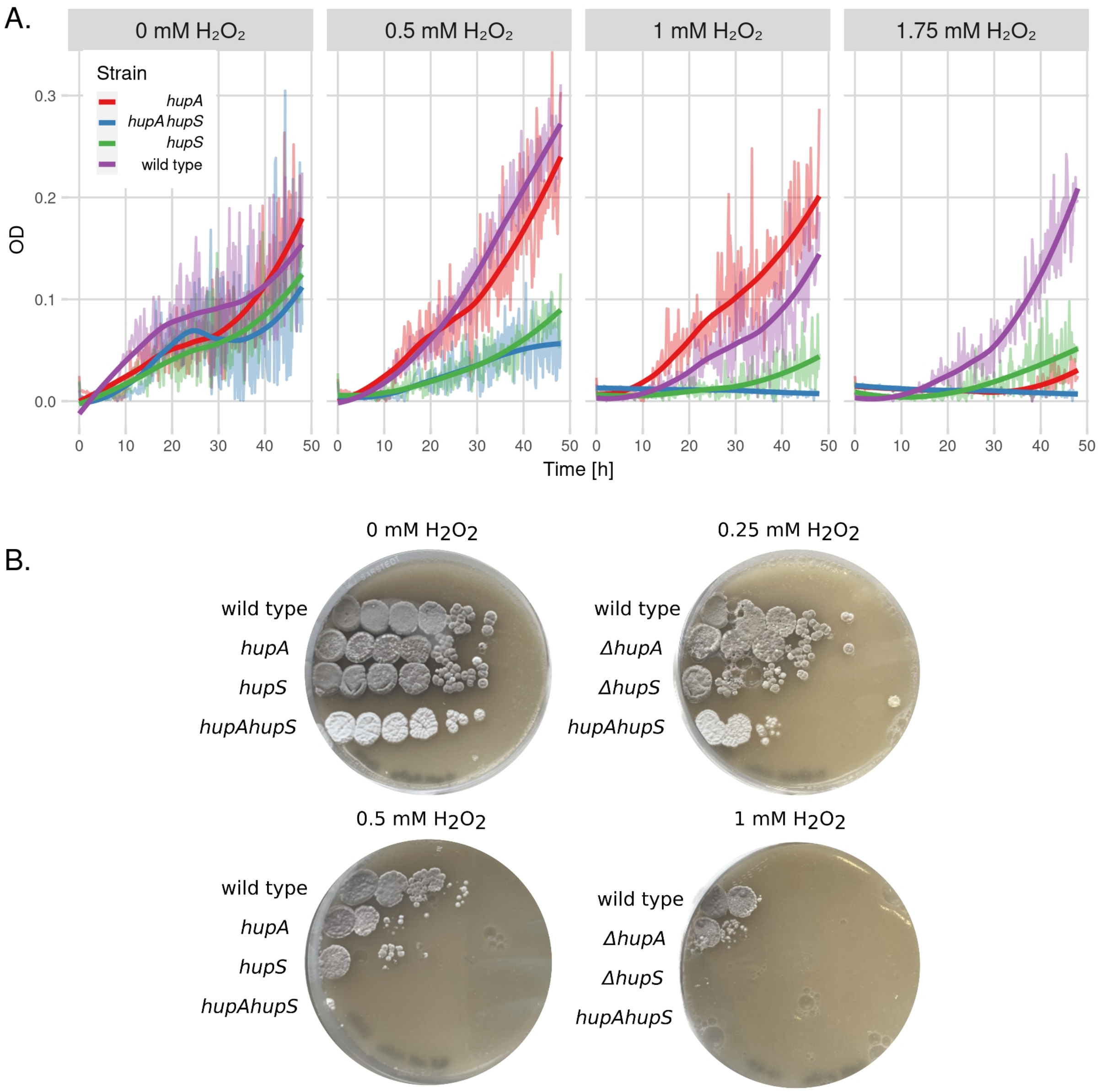
A.
0 mM H2O2
0.5 mM H2O2
1 mM H2O2
1.75 mM H2O2
Strain
hupA
hupA hupS
hupS
wild type
OD
0.0
0.1
0.2
0.3
0
10
20
30
40
50
Time [h]
B.
0 mM H2O2
0.25 mM H2O2
0.5 mM H2O2
1 mM H2O2
wild type
hupA
hupS
hupAhupS
ΔhupA
ΔhupS

Figure S2

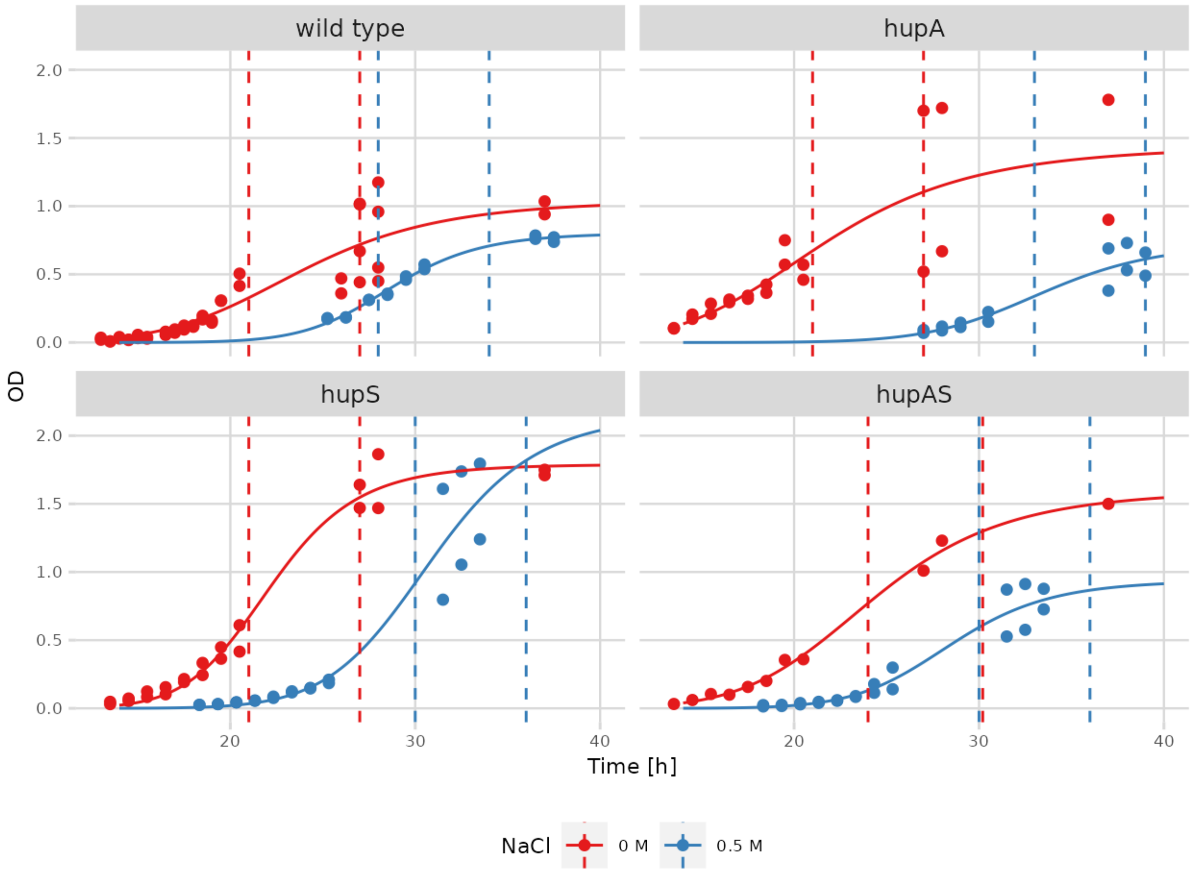
wild type
hupA
hupS
hupAS
OD
Time [h]
2.0
1.5
1.0
0.5
0.0
20
30
40
NaCl
0 M
0.5 M

Figure S3

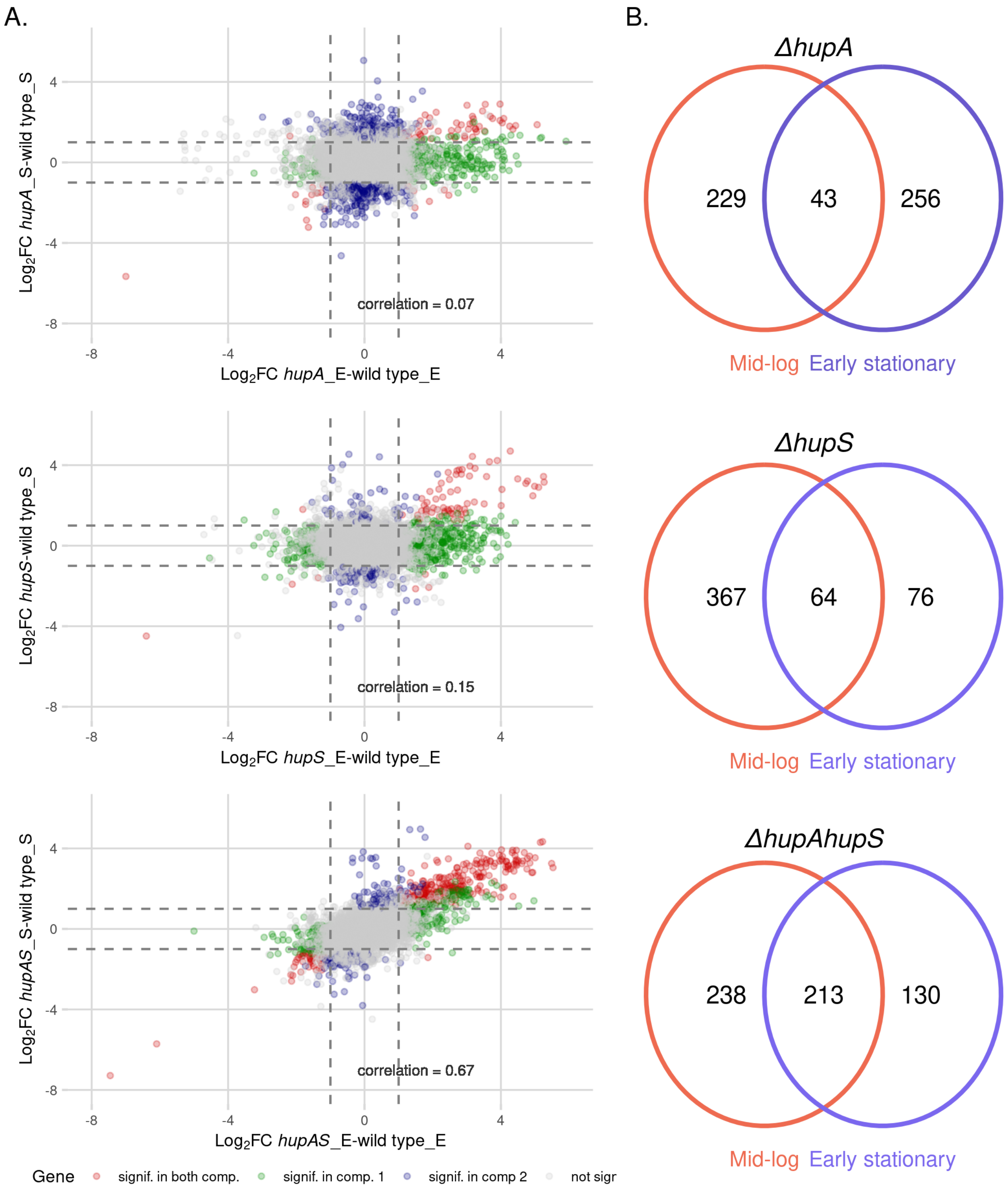
A.
B.
correlation = 0.07
Log2FC hupA_E-wild type_E
Log2FC hupA_S-wild type_S
correlation = 0.15
Log2FC hupS_E-wild type_E
Log2FC hupS-wild type_S
correlation = 0.67
Log2FC hupAS_E-wild type_E
Log2FC hupAS_S-wild type_S
Gene
signif. in both comp.
signif. in comp. 1
signif. in comp 2
not sigr
ΔhupA
229
43
256
ΔhupS
367
64
76
ΔhupAhupS
238
213
130
Mid-log
Early stationary

Figure S4

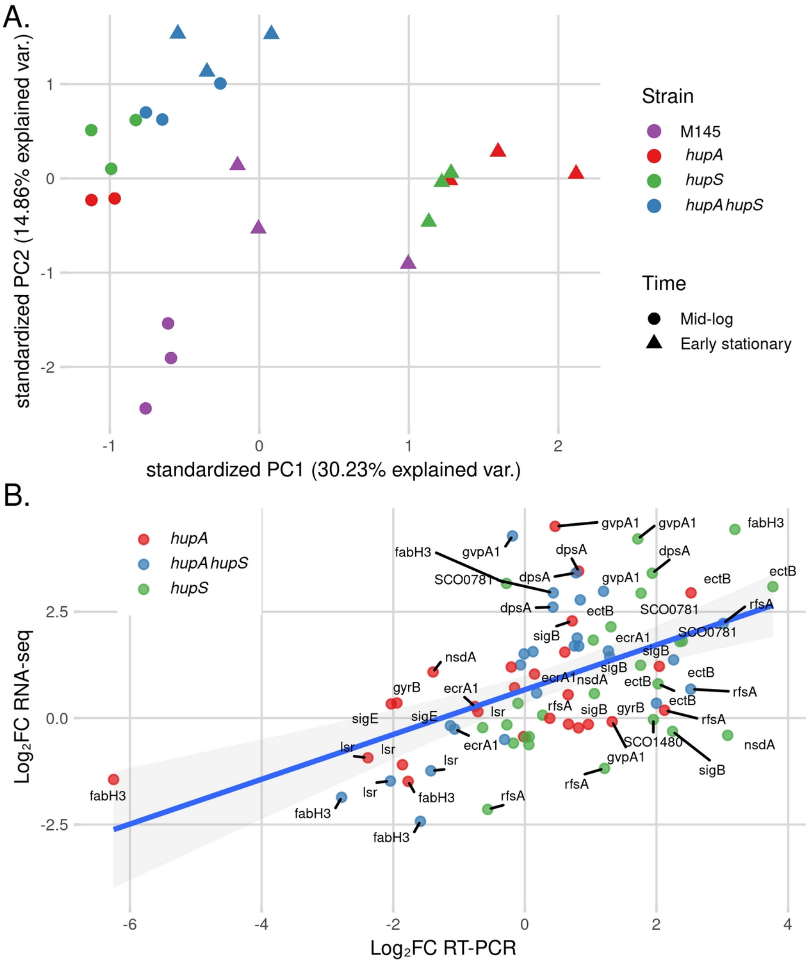

C.

gene	*hupA* E	*hupA* S	*hupS* E	*hupS* S	*hupAhupS* E	*hupAhupS* S	gene	*hupA* E	*hupA* S	*hupS* E	*hupS* S	*hupAhupS* E	*hupAhupS* S
SCO0781*	1.20	NA	2.93	1.81	2.94	1.88	lsr*	NA	NA	NA	NA	-1.24	-1.48
SCO7328	1.51	NA	NA	NA	1.76	1.98	nsdA*	NA	NA	NA	NA	1.58	1.69
argR	NA	NA	NA	NA	NA	2.69	osdR	2.65	NA	2.07	NA	2.50	NA
catB	2.74	1.62	2.37	NA	3.03	4.10	redD	NA	NA	1.92	2.69	NA	NA
cvnA12	NA	NA	5.25	3.44	4.69	3.79	rfsA*	NA	NA	NA	NA	NA	2.23
dpsA*	3.45	NA	3.40	NA	3.41	2.61	rsbV	3.12	NA	2.90	NA	2.87	2.39
ecrA1*	NA	NA	1.83	2.15	NA	NA	sIHF	1.55	NA	1.24	NA	1.70	1.56
ectB*	2.94	1.22	3.09	NA	1.37	2.78	sapA	NA	NA	NA	NA	NA	-2.38
fabH3*	NA	NA	3.16	4.43	NA	NA	sigB*	2.28	NA	1.80	NA	1.25	1.44
gvpA1*	4.51	NA	4.21	NA	4.28	2.98	sigK	4.04	1.97	2.83	NA	4.38	3.21
gyrA	1.46	NA	NA	NA	1.03	1.95	sigL	2.55	NA	2.32	NA	2.32	1.96
gyrB*	NA	NA	NA	NA	NA	1.51	sigM	5.06	1.87	3.51	NA	4.41	3.65

* - confirmed with RT-PCR, E - Mid-log, S - Early stationary

Figure S5

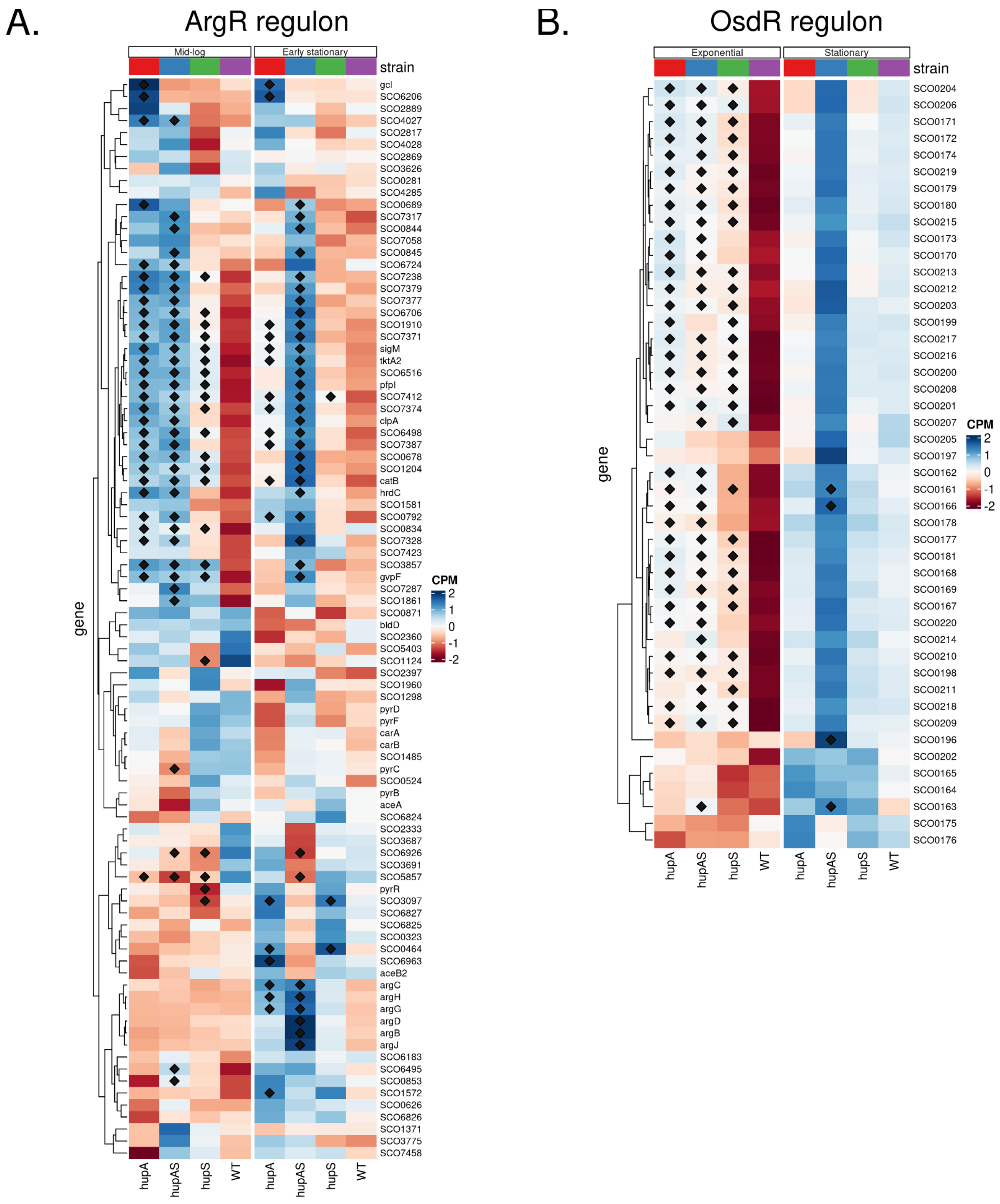

A.
ArgR regulon
Mid-log
Early stationary
strain
gene
gcl
SCO6206
SCO2889
SCO4027
SCO2817
SCO4028
SCO2869
SCO3626
SCO0281
SCO4285
SCO0689
SCO7317
SCO0844
SCO7058
SCO0845
SCO6724
SCO7238
SCO7379
SCO7377
SCO6706
SCO1910
SCO7371
sigM
tktA2
SCO6516
pfpl
SCO7412
SCO7374
clpA
SCO6498
SCO7387
SCO0678
SCO1204
catB
hrdC
SCO1581
SCO0792
SCO0834
SCO7328
SCO7423
SCO3857
gvpF
SCO7287
SCO1861
SCO0871
bldD
SCO2360
SCO5403
SCO1124
SCO2397
SCO1960
SCO1298
pyrD
pyrF
carA
carB
SCO1485
pyrC
SCO0524
pyrB
aceA
SCO6824
SCO2333
SCO3687
SCO6926
SCO3691
SCO5857
pyrR
SCO3097
SCO6827
SCO6825
SCO0323
SCO0464
SCO6963
aceB2
argC
argH
argG
argD
argB
argJ
SCO6183
SCO6495
SCO0853
SCO1572
SCO0626
SCO6826
SCO1371
SCO3775
SCO7458
CPM
2
1
0
-1
-2
hupA
hupAS
hupS
WT
hupA
hupAS
hupS
WT
B.
OsdR regulon
Exponential
Stationary
strain
gene
SCO0204
SCO0206
SCO0171
SCO0172
SCO0174
SCO0219
SCO0179
SCO0180
SCO0215
SCO0173
SCO0170
SCO0213
SCO0212
SCO0203
SCO0199
SCO0217
SCO0216
SCO0200
SCO0208
SCO0201
SCO0207
SCO0205
SCO0197
SCO0162
SCO0161
SCO0166
SCO0178
SCO0177
SCO0181
SCO0168
SCO0169
SCO0167
SCO0220
SCO0214
SCO0210
SCO0198
SCO0211
SCO0218
SCO0209
SCO0196
SCO0202
SCO0165
SCO0164
SCO0163
SCO0175
SCO0176
CPM
2
1
0
-1
-2
hupA
hupAS
hupS
WT
hupA
hupAS
hupS
WT

Figure S6

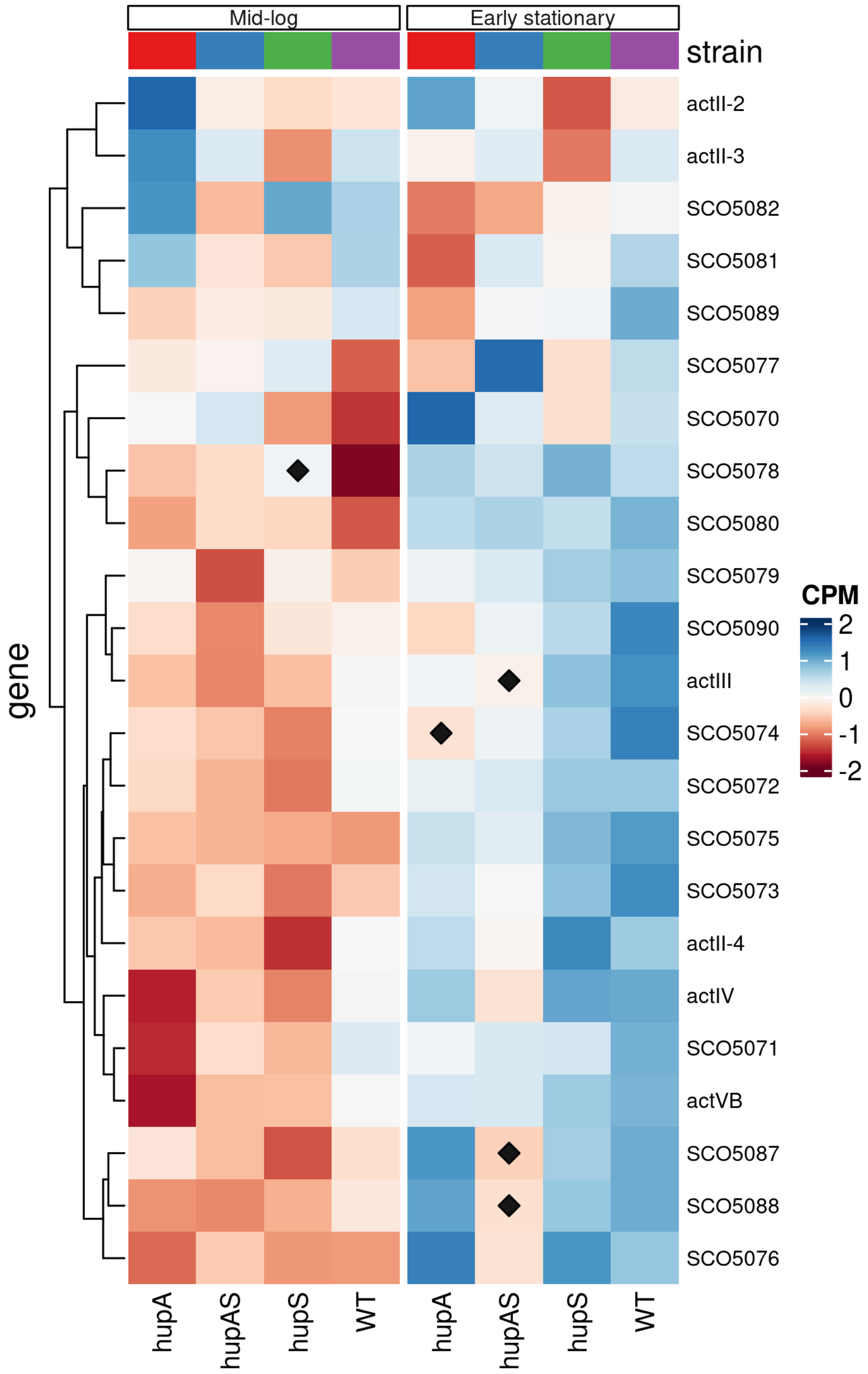
Mid-log
Early stationary
strain
gene
actII-2
actII-3
SCO5082
SCO5081
SCO5089
SCO5077
SCO5070
SCO5078
SCO5080
SCO5079
SCO5090
actIII
SCO5074
SCO5072
SCO5075
SCO5073
actII-4
actIV
SCO5071
actVB
SCO5087
SCO5088
SCO5076
CPM
2
1
0
-1
-2
hupA
hupAS
hupS
WT
hupA
hupAS
hupS
WT

Figure S7

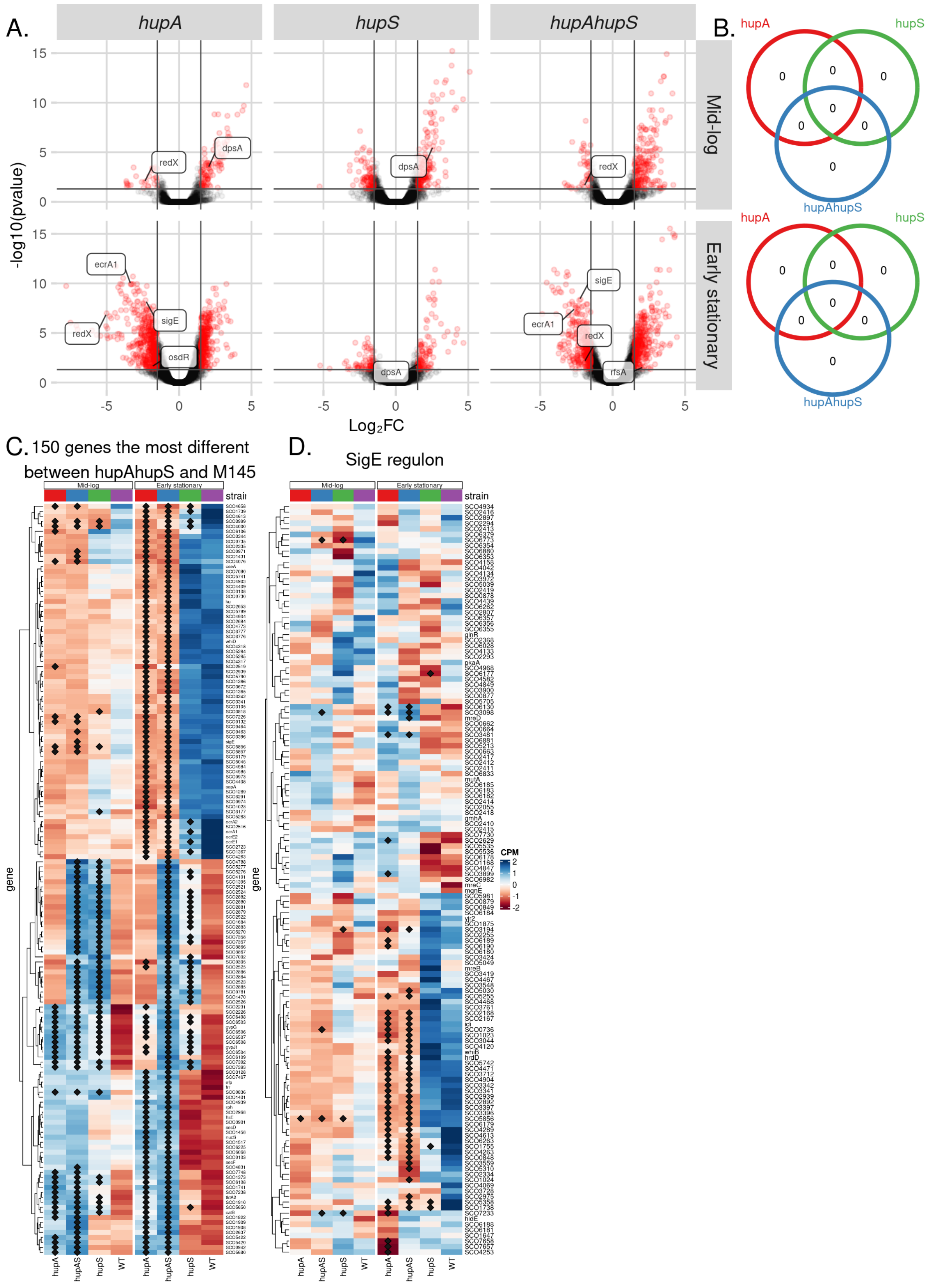
A.
hupA
hupS
hupAhupS
B.
Mid-log
Early stationary
-log10(pvalue)
Log2FC
redX
dpsA
ecrA1
sigE
osdR
rfsA
C. 150 genes the most different between hupAhupS and M145
D. SigE regulon
Mid-log
Early stationary
strain
gene
CPM
hupA
hupAS
hupS
WT

Figure S8

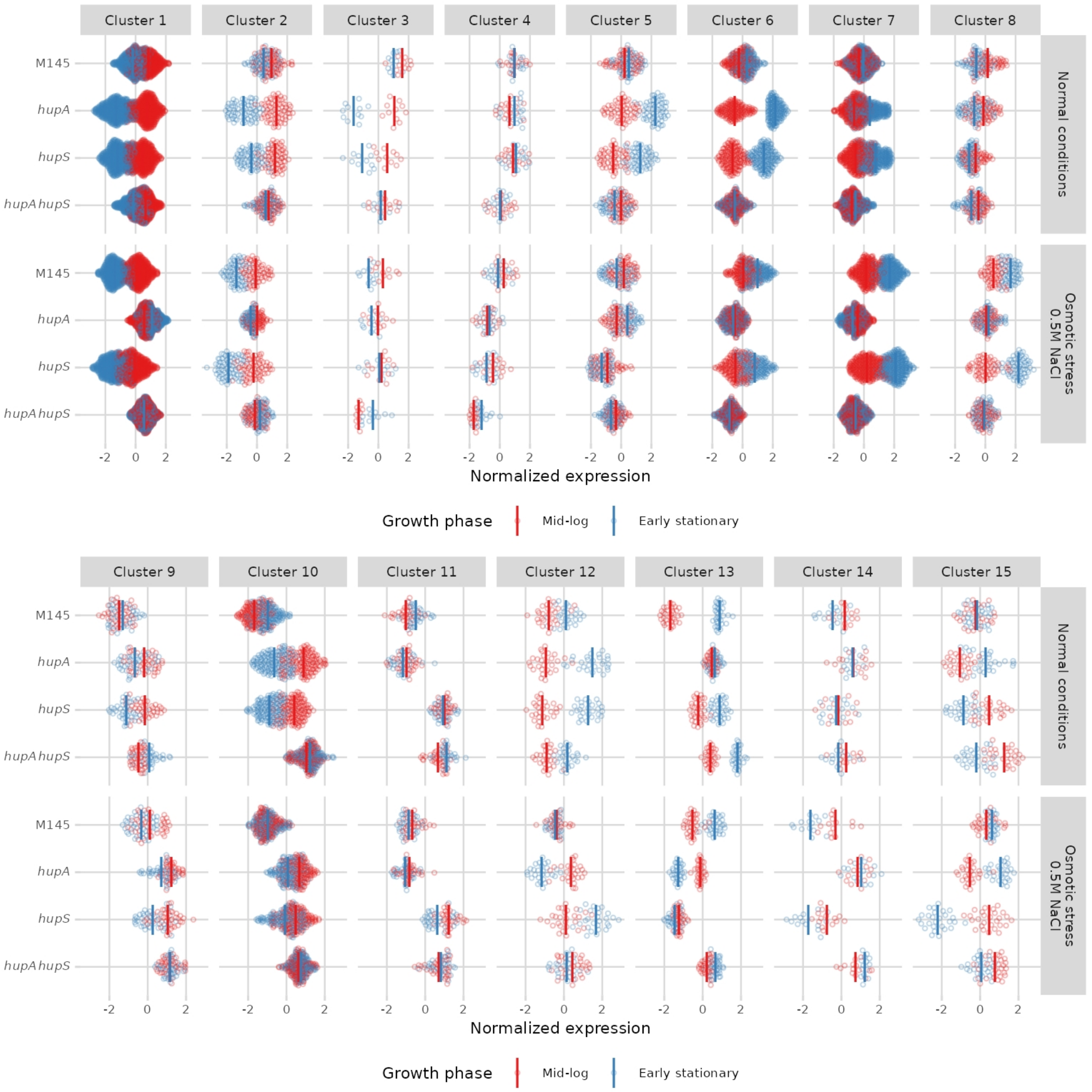
Cluster 1
Cluster 2
Cluster 3
Cluster 4
Cluster 5
Cluster 6
Cluster 7
Cluster 8
Cluster 9
Cluster 10
Cluster 11
Cluster 12
Cluster 13
Cluster 14
Cluster 15
M145
hupA
hupS
hupA hupS
Normal conditions
Osmotic stress 0.5M NaCl
-2
0
2
Normalized expression
Growth phase
Mid-log
Early stationary

Figure S9

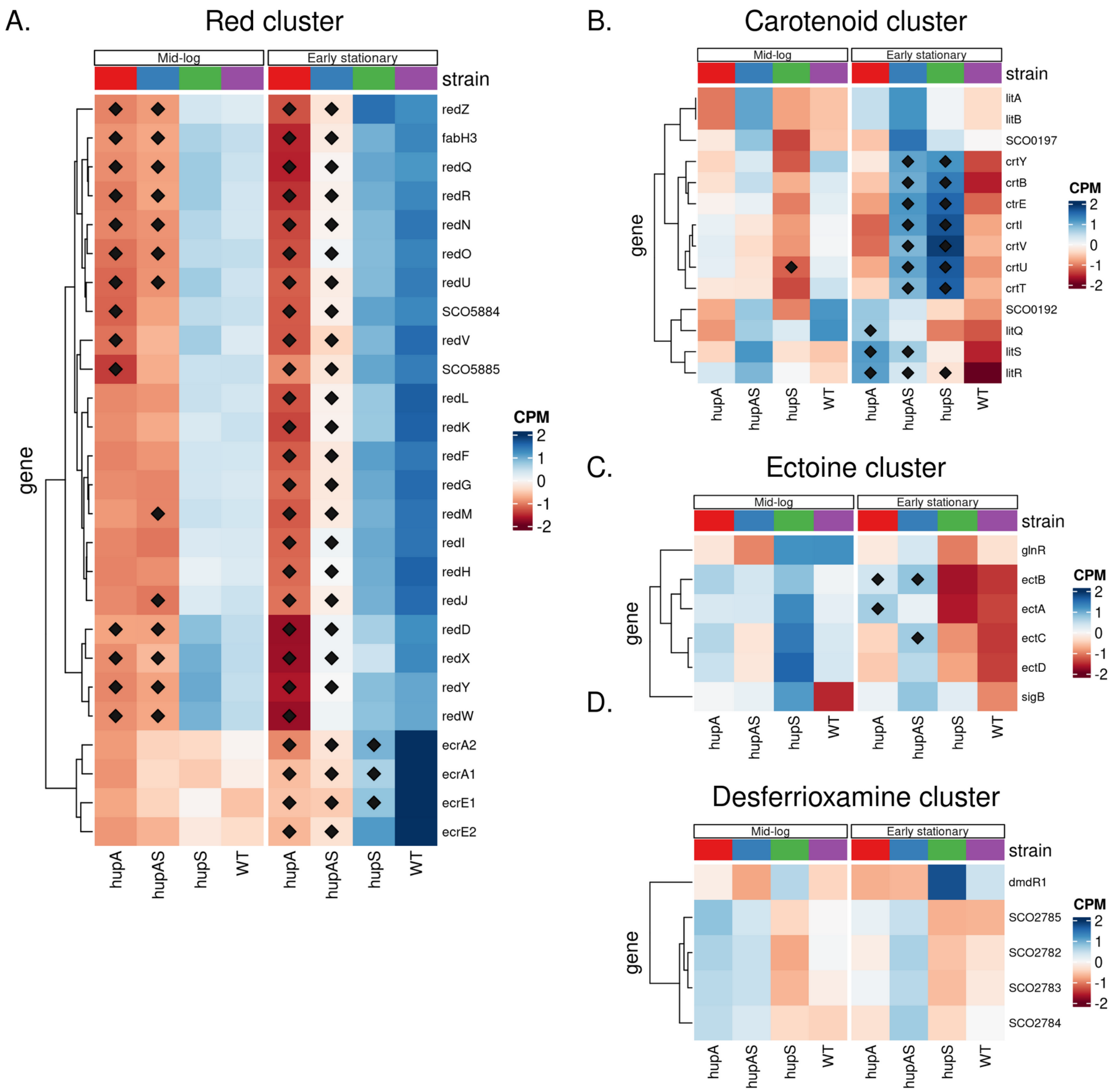
A.
Red cluster
Mid-log
Early stationary
strain
gene
CPM
2
1
0
-1
-2
redZ
fabH3
redQ
redR
redN
redO
redU
SCO5884
redV
SCO5885
redL
redK
redF
redG
redM
redI
redH
redJ
redD
redX
redY
redW
ecrA2
ecrA1
ecrE1
ecrE2
hupA
hupAS
hupS
WT
B.
Carotenoid cluster
litA
litB
SCO0197
crtY
crtB
ctrE
crtI
crtV
crtU
crtT
SCO0192
litQ
litS
litR
C.
Ectoine cluster
glnR
ectB
ectA
ectC
ectD
sigB
D.
Desferrioxamine cluster
dmdR1
SCO2785
SCO2782
SCO2783
SCO2784

Figure S10
